# Onion‐Mitochondria Inhibit Lipopolysaccharide‐Induced Acute Lung Injury by Shaping Lung Macrophage Mitochondrial Function

**DOI:** 10.1002/advs.202506107

**Published:** 2025-10-06

**Authors:** Qingbo Xu, Yun Teng, Yinan Huang, Jingyao Mu, Lucy Teng, Hongjia Qian, Qiming Huang, Minmin Liu, Yi Zou, Lifeng Zhang, Michael L Merchant, Xiang Zhang, Jun Yan, Huang‐Ge Zhang

**Affiliations:** ^1^ Robley Rex Department Veterans Affairs Medical Center Louisville KY 40206 USA; ^2^ Department of Microbiology and Immunology University of Louisville Louisville KY USA; ^3^ Brown Cancer Center Department of Medicine University of Louisville School of Medicine Louisville KY 40202 USA; ^4^ Kidney Disease Program and Clinical Proteomics Center University of Louisville Louisville KY USA; ^5^ Department of Pharmacology and Toxicology University of Louisville Louisville KY 40202 USA

**Keywords:** Cardiolipin peroxidation, Complex I subunits NADH dehydrogenase 1 (ND1), Dynamin‐related protein 1 (DRP1) phosphorylation, Mitochondrial dysfunction, Plant mitochondria‐animal mitochondria cross‐kingdom fusion

## Abstract

Mitochondrial dysfunction contributes to various inflammatory‐related diseases by triggering the release of inflammatory molecules. Targeting mitochondrial dysfunction is emerging as a promising avenue for treating inflammatory diseases. Here, it is demonstrated that dietary plant‐derived mitochondria (P‐Mit) are capable of rescuing the lung macrophage mitochondrial (M‐Mit) dysfunction in lipopolysaccharide (LPS)‐induced mouse acute lung injury (ALI). Specifically, oral administration of dietary onion‐derived mitochondria (O‐Mit) can travel from the gut to the lungs in ALI mice, where preferentially uptake by lung macrophage mediated by the interaction between O‐Mit phosphatic acid (PA) and macrophage complement C3b/C4b receptor 1 Like (CR1L), followed by fusing with murine M‐Mit and by reprograming the M‐Mit energy metabolism in the lungs of ALI mice. Further evidence suggests that O‐Mit enriches methyl 3,4‐dihydroxybenzoate (MDHB) inhibits M‐Mit NADH dehydrogenase subunit 1 (ND1) gene expression in the epigenetic process, which represses LPS‐induced complex I‐related oxidative stress activation and excessive mitochondrial fission via modulating dynamin‐related protein 1 (DRP1) phosphorylation and cardiolipin peroxidation in M‐Mit, eventually rescues the LPS‐induced ALI. Given LPS‐induced mouse model of ALI is widely used to study human ALI and acute respiratory distress syndrome, this finding provides a clinical potential for the treatment of human ALI via edible P‐Mit.

## Graphical abstract

Proposed model of orally administered onion mitochondria (O‐Mit) uptake by lung macrophages and fuse with macrophage mitochondria (M‐Mit). This fusion reprograms the metabolism of dysfunctional M‐Mit in lipopolysaccharide‐induced murine acute lung injury by modulating dynamin‐related protein 1 (DRP1) phosphorylation and cardiolipin peroxidation, thereby alleviating lung inflammation. These findings reveal a previously unexplored strategy using plant mitochondria in mitochondrial transfer therapy.

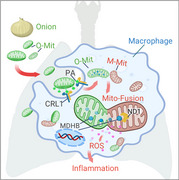

## 1 Introduction

An emerging body of literature indicates that intercellular mitochondrial transfer regulates the physiology and function of various organ systems in health and disease and has therapeutic applications.^[^ ^1^, ^2^ ^]^ However, mitochondrial transfer therapy faces several challenges.^[^ ^3^, ^4^, ^5^ ^]^ First, mammalian cell‐derived mitochondria can cause immune reactions in the recipient. Second, mammalian cell‐derived mitochondria require optimization for each batch, including donor screening, function evaluation, and histocompatibility. Limited tissue availability necessitates multiple propagations, leading to variable quality and therapeutic effects. In addition, targeting the tissue where the pathological effect takes place is challenging. Furthermore, there are ethical and regulatory challenges for mammalian mitochondria transfer therapy, especially when it comes to human applications. The long‐term effects and potential risks require thorough evaluation before widespread clinical use.

Food immune tolerance is well documented. Plants in our diet contain mitochondria, and the fundamental structure and function of mitochondria are conserved across plants and animals. Both plant and animal mitochondria share major regulatory, bioenergetic, and chemical substrate pathways related to ancestral endosymbiotic relationships.^[^ ^6^ ^]^ Mitochondria are dynamic organelles that constantly change shape through two key processes: fusion and fission. Fusion is the process where two or more mitochondria merge to form a single, larger mitochondrion. This helps in combating stress as a result of stimulation that can be caused by events such as bacterial released lipopolysaccharide (LPS).^[^ ^7^ ^]^ By merging mitochondria can share their contents, which helps in repairing damage and maintaining function. Not only does fusion of mitochondria occur in animal cells, but it has also been demonstrated in plants such as onions.^[^ ^8^ ^]^ However, whether plant‐to‐mammalian intercellular mitochondrial transfer occurs, and how it affects the recipient cell's function, remains unknown. Answering these questions could fundamentally alter our understanding as to how mammalian mitochondrial biology can be regulated by plant mitochondria (P‐Mit). This knowledge could in turn lead to plant mitochondrial transfer therapy for preventing or treating human diseases.

In this study, we demonstrate that orally administered onion‐derived mitochondria (O‐Mit) can traffic from the gut to the lung and fuse with macrophage mitochondria (M‐Mit). O‐Mit methyl 3,4‐dihydroxybenzoate (MDHB) is then incorporated into M‐Mit and reverses the LPS‐mediated M‐Mit dysfunction and LPS‐induced overproduction of interleukin‐1 beta (IL‐1β), interferon gamma (IFN‐γ), interleukin 6 (IL‐6), guanosine‐5′‐triphosphate (GTP), glutathione disulfide (GSSG), and mitochondrial superoxide. Collectively, oral administration of O‐Mit inhibits LPS‐induced acute lung injury (ALI) by modulating M‐Mit energic metabolic and dynamic activities. These findings highlight a previously unknown function of P‐Mit in cross‐kingdom fusing and shaping mammalian mitochondrial metabolic pathways in lung inflammation and have implications for developing plant mitochondrial‐based transfer therapy.

## 2 Results

### 2.1 Onion Mitochondria Transfer Therapy Inhibits LPS‐Induced Acute Lung Injury (ALI) in Mice

Intercellular mitochondrial transfer has significant implications for lung inflammation.^[^ ^9^ ^]^ Recent studies have shown that this transfer can occur through a variety of mechanisms.^[^ ^10^, ^11^, ^12^, ^13^, ^14^, ^15^, ^16^ ^]^ Stressed epithelial cells in the lungs can receive healthy mitochondria by mitochondrial transfer therapy. This process has been observed to reduce inflammatory cell infiltration, collagen deposition, and mucus hypersecretion in the lungs.^[^ ^17^ ^]^ But mitochondrial therapy faces many challenges including eliciting immune responses.^[^ ^18^, ^19^ ^]^ Diets including edible plant‐inducing oral immune tolerance are well‐known.^[^ ^20^, ^21^ ^]^ Therefore, we hypothesized that edible plant‐derived mitochondria (P‐Mit) can be potentially used for mitochondrial transfer therapy. As proof of concept, mitochondria isolated from onion (O‐Mit), soybean (S‐Mit), and garlic (G‐Mit) were used to test whether edible P‐Mit can protect against lipopolysaccharide (LPS)‐induced inflammation in mice. LPS released from bacteria has been known to induce ALI.^[^ ^22^ ^]^ To extract mitochondria from onions, soybeans, and garlic, we used differential centrifugation followed by a sucrose density gradient method as described in previous studies.^[^ ^23^, ^24^, ^25^, ^26^ ^]^ Purified P‐Mit were collected from the band at the 1.2 m sucrose layer (Figure S1A, Supporting Information). The yields of O‐Mit, S‐Mit, and G‐Mit were 11.4 ± 1.02, 9.40 ± 1.52, and 10.9 ± 1.52 mg per gram of tissues, respectively (Figure S1B, Supporting Information). Transmission electron microscopy (TEM) shows that the P‐Mit were short rod‐shaped or spherical, with a double membrane structure. The outer membrane of the P‐Mit was smooth, and the inner membrane formed inward folds, known as cristae (Figure S1C, Supporting Information). The analysis of length indicated that P‐Mit ranged from 1 to 2 µm and O‐Mit exhibits the largest length compared G‐Mit and S‐Mit (Figure S1D, Supporting Information). The zeta potential of the P‐Mit ranged from −21 to −25 mV (Figure S1E, Supporting Information). A refractometer was used to measure the refractive index (RI) of P‐Mit at different concentrations, and the results demonstrated that the RI increased with an increasing concentration of P‐Mit and different P‐Mit showed no difference in RI (Figure S1F, Supporting Information). The lipid profile analysis of P‐Mit suggested a diversity in the lipid composition in P‐Mit. O‐Mit was highest in phosphatidic acid (PA), S‐Mit was highest in phosphatidylethanolamine (PE), and G‐Mit was highest in digalactosyldiacylglycerol (MGDG) (Figure S1G, Supporting Information). MGDG is a galactolipid, specifically abundant in photosynthetic membranes of plant leaves.^[^ ^27^ ^]^ Complex I is a crucial enzyme that initiates the electron transport chain within mitochondria. Complex I activity test indicated that O‐Mit has the highest activity compared to G‐Mit and S‐Mit (Figure S1H, Supporting Information). The Complex I activity of all P‐Mit was eliminated by boiling at 100 °C for 5 min (Figure S1H, Supporting Information). Inflammation‐activated macrophage triggers the adaptive immune response and releases pro‐inflammatory factors in lung inflammatory processes. To test if P‐Mit reduces macrophage‐based inflammation, we investigated the effect of P‐Mit in a LPS‐induced inflammation in macrophage IC‐21 cells. The results suggested that the expression of LPS induced interferon gamma (IFN‐γ) (Figure S2A, Supporting Information) and IL‐1β (Figure S2B, Supporting Information), and each was significantly decreased by O‐Mit in a dose‐dependent manner. G‐Mit and S‐Mit had no effect on IFN‐γ or IL‐1β. This data provided the impetus for using O‐Mit in vivo studies.

First, we tested the integrity and stability of O‐Mit in a gastrointestinal environment. To mimic the gastrointestinal environment, O‐Mit labeled with mitochondrial potential dye JC‐1 was incubated with gastric fluid or gut fluid isolated from the stomach and small intestine for 2 h at 37 °C. Flow cytometry (FACS) and PCR analysis were performed to assess the JC‐1 intensity and mitochondrial DNA (mtDNA) nad1 of O‐Mit, respectively. The FACS analysis shows that JC‐1 labeled O‐Mit were not shifted from the red fluorescence (PE channel (polarized) to the green fluorescence (FITC channel (depolarized) in gastric fluid or gut fluid (Figure S3A, Supporting Information, left panel). The oxygen flux assay shows no significant impact of oxygen consumption on O‐Mit exposed to gastric fluid and gut fluid (Figure S3B, Supporting Information, left panel). These data indicated that O‐Mit was functional and intact under gastric fluid and gut fluid conditions. PCR analysis also suggested little influence of gastric and gut fluid on O‐Mit mtDNA integrity (Figure S3C, Supporting Information, left panel). We further determined whether O‐Mit lipid integrity plays a role in maintaining stability. O‐Mit and total lipids extracted from O‐Mit were incubated with gastric and gut fluid for 2 h at 37 °C. The lipids were extracted followed by a thin‐layer chromatography (TLC) analysis. TLC results indicated that lipids presented in the context of O‐Mit are stable and lipids extracted from disrupted O‐Mit were susceptible to being degraded by gastric and gut fluid (Figure S3D, Supporting Information). Since mitochondria are present in both plant cells as well mammalian cells but with different molecular structures,^[^ ^28^, ^29^ ^]^ we also compared the stability of P‐Mit and mammalian mitochondria. The mammalian mitochondria were isolated from mouse gut epithelial cells (E‐Mit) and labeled with JC‐1 followed by incubation with gastric and gut fluid. The results showed that gastric and gut fluid increased the green fluorescence and decreased the red fluorescence in E‐Mit, indicating that JC‐1‐based mitochondria membrane potential was reduced (Figure S3A, Supporting Information, right panel). Gastric and gut fluid also reduced oxygen flux (Figure S3B, Supporting Information, right panel), mtDNA (Figure S3C, Supporting Information, right panel), and lipid composition (Figure S3D, Supporting Information) of E‐Mit, indicating P‐Mit are functional and intact under the gastrointestinal environment. Given that plant‐derived foods are known to promote immune tolerance by activating regulatory T cells (Tregs) in the gut,^[^ ^20^, ^21^ ^]^ we investigated the immunomodulatory effects of P‐Mit on Tregs. Mice were administered P‐Mit via oral gavage, and after 6 h, leukocytes were isolated from the small intestine for flow cytometric analysis. Our results demonstrated that O‐Mit significantly induced the expression of IL‐10 (Figure S4A, Supporting Information) and TGF‐β (Figure S4B, Supporting Information) in FoxP3⁺ CD4⁺ T cells.

To elucidate the cellular mechanism underlying this induction, we first identified the primary target cells of O‐Mit. Confocal microscopy revealed that O‐Mit was taken up by F4/80⁺ macrophages, but not by CD4⁺ T cells (Figure S4C, Supporting Information). Further analysis showed that O‐Mit treatment increased TGF‐β expression and reduced reactive oxygen species (ROS) levels in intestinal macrophages (Figure S4D, Supporting Information). Given that macrophages regulate Treg activity through TGF‐β secretion and ROS modulation,^[^ ^30^, ^31^ ^]^ we treated gut‐derived macrophages with O‐Mit and collected the conditioned medium for co‐culture with FACS‐sorted intestinal FoxP3⁺CD4⁺ Treg cells. The conditioned medium from O‐Mit‐treated macrophages significantly enhanced IL‐10 and TGF‐β expression in Treg cells. Importantly, neutralization of TGF‐β with a specific antibody prior to co‐culture abolished this effect (Figure S4E, Supporting Information), indicating that O‐Mit promotes Treg activity indirectly by modulating macrophage function specifically through stimulation of TGF‐β production and suppression of oxidative stress. Collectively, our data indicate that macrophage‐derived TGF‐β is a key mediator of O‐Mit‐induced Treg activation.

Since P‐Mit is stable in the gut, to determine the distribution of P‐Mit after oral administration, we generated an ALI model in C57BL/6J (B6) mice by administering LPS (50 µg mice^−1^) intranasally.^[^ ^32^ ^]^ Six hours after the administration of LPS, fluorescence dye DiR‐labeled O‐Mit, S‐Mit, and G‐Mit were orally gavage‐given to the ALI mice. Two hours after the gavage, the ALI mice organs were collected and scanned using an Odyssey imager. The fluorescent imaging results showed that O‐Mit exhibited the highest accumulation in the lungs of the ALI mice compared to S‐Mit and G‐Mit (FigureS5A,B, Supporting Information). Different types of P‐Mit have no different distribution in other tissues such as liver, heart, and brain (Figure S5A,B, Supporting Information). To test the dynamic distribution in circulation, the O‐Mit/DiR signal in peripheral blood analysis was conducted, and the result suggested that O‐Mit/DiR can be detected in blood as early as 30 min post gavage (Figure S5C, Supporting Information), reaching a peak at 2 h and started to decline after 6 h (Figure S5C, Supporting Information). O‐Mit was observed to have the greatest heterogeneity in size compared to S‐Mit and G‐Mit (Figure S1C,D, Supporting Information). To test whether a subset of the sizes of O‐Mit preferentially distributes to the lungs of ALI mice, the O‐Mit isolated from the band at 1.2 m after sucrose gradient (0.6 to 1.8 m) centrifugation (Figure S1A, Supporting Information) were reloaded in a sucrose gradient (1 to 1.25 m) (Figures S5D, Supporting Information) to separate the O‐Mit based on size variation. Two different sizes of O‐Mit were obtained from the second sucrose gradient centrifugation at 12 000 × g. The average sizes of large (2.38 ± 0.32 µm) and small (0.63 ± 0.12 µm) O‐Mit were determined by TEM analysis (Figures S5D, Supporting Information). The two different sizes of O‐Mit were labeled with DiR dye and orally gavage‐given in the same amounts to LPS‐induced ALI mice. Image analysis suggested that small O‐Mit was preferentially retained in the liver, and the large size O‐Mit tended to be trapped in the lungs (Figures S5E, Supporting Information). To test if intranasal administration‐induced lung inflammation enhances P‐Mit retention in the lungs, we estimated the distribution of P‐Mit/DiR in naïve mice without LPS treatment. Our image analysis showed that there is no difference in lung and liver distribution without LPS stimulation (Figure S5F, Supporting Information) compared with LPS stimulation (Figure S5A,B, Supporting Information), suggesting that LPS‐induced ALI enhances P‐Mit retention in the lungs. To assess the in vivo stability of O‐Mit in lungs, lung bronchoalveolar lavage fluid (BALF) was collected from the ALI mice fed with O‐Mit. Mitochondria in the BALF were isolated, and plant‐derived mitochondrial exclusive protein isocitrate dehydrogenase 3 (IDH3) was tested using a western blot. The results suggested that 9.17 ± 0.31% and 7.23 ± 0.32% of O‐Mit IDH3 was detected in the lungs at 1 and 2 h after gavage, respectively, when compared to the total input amount administered (Figure S6A, Supporting Information). These results were consistent with the analysis of mtDNA nad1 in O‐Mit using qPCR (Figure S6B, Supporting Information), the fluorescent dye intensity analysis using O‐Mit/DiO (Figure S6C, Supporting Information) and mitochondrial membrane potential analysis using O‐Mit/JC‐1 fluorescence indicator at 590 nm (red, polarized) and 529 nm (green, depolarized) (Figure S6D, Supporting Information, left panel). However, there was no change in the ratio of JC‐1 fluorescence at 590/529 nm in the lungs, indicating that the O‐Mit membrane potential activity is stable in the lung microenvironment (Figure S6D, Supporting Information, right panel).

**Figure 1:**
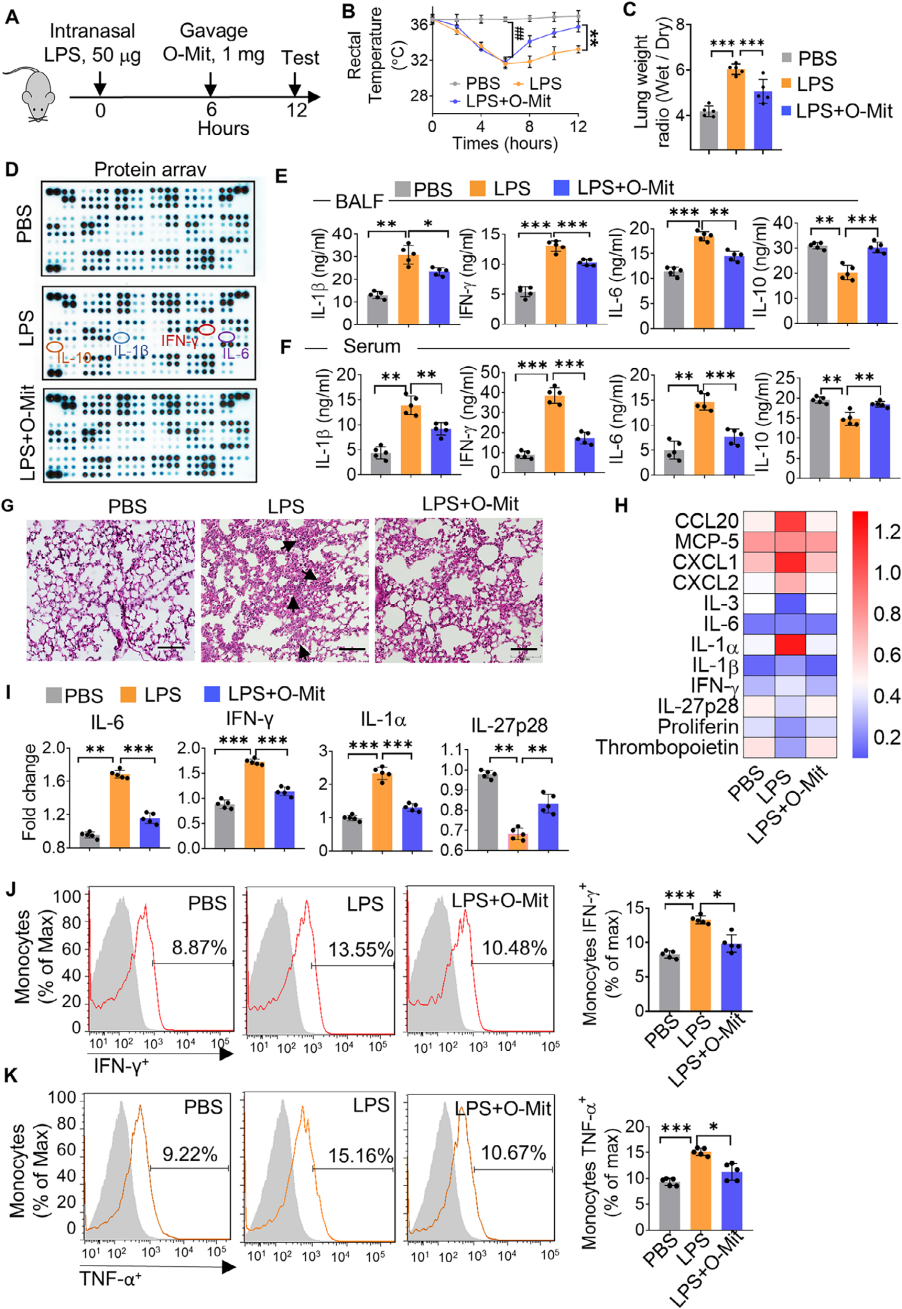
O‐Mit can suppress LPS‐induced acute lung injury (ALI) in mice. A) Schematic illustration of the acute lung injury (ALI) model generated in mice. The C57BL/6J (B6) mice were administered LPS (50 µg each^−1^, *n* = 5) via intranasal route. Six hours later the mice were administered with O‐Mit via oral gavage (1 mg each^−1^, *n *= 5). Six hours post gavage, the mice were euthanized and subjected to testing. B) Rectal temperatures of mice were recorded every 2 h after intranasal LPS. At 6 h, a significant difference was observed in the LPS group versus the PBS group (^##^, *p* < 0.01). O‐Mit (1 mg mouse^−1^, *n* = 5) was given orally at 6 h. After 6 h, O‐Mit‐treated mice showed a significant temperature difference versus the LPS group (**, *p* < 0.01). (two‐tailed *t*‐test). C) The lung wet/dry weight ratio was used to assess pulmonary edema in mice (*n *= 5) from different treatment groups. D) Cytokines in lung tissues from ALI mice (*n* = 5) in different treatment groups were analyzed using the Proteome Profiler Mouse XL Cytokine Array. The blots indicated that O‐Mit reverses the impact of LPS on the expression of cytokines IL‐10, IL‐1β, IFN‐γ, and IL‐6 in the lungs. E,F) ELISA analysis of cytokines IL‐1β, IFN‐γ, IL‐6, and IL‐10 in bronchoalveolar lavage fluid (BALF) (E) and serum (F) from mice in different treatment groups. G) Lung tissue of mice paraffin‐embedded, sectioned, and stained with hematoxylin and eosin (H&E). Original magnification X20. Bar scale 100 µm. The arrows indicate the infiltration of inflammatory cells in the alveolar cavity and interstitial areas, as well as the thickening of the alveolar wall. H) Heatmaps show cytokine gene expression in lung tissue detected using a q‐PCR array kit (QIAGEN). The results indicate differences in cytokine expression among mice from different treatment groups. I) Individual q‐PCR analysis of IL‐6, IFN‐γ, IL‐1α, and IL‐27p28 cytokine genes in lung tissue (*n* = 5). J,K) FACS analysis of cytokines IFN‐γ (J) and TNF‐α (K) in lung monocytes of ALI mice with or without O‐Mit gavage (*n* = 5). Differences between percentages were analyzed with a chi‐square test. Differences between individual groups were analyzed via a two‐way ANOVA test. Data are representative of three independent experiments (error bars, standard deviation (SD)). * *p* < 0.05, ** *p* < 0.01, *** *p* < 0.001.

Given that O‐Mit is capable of inhibiting the expression of LPS‐induced inflammatory cytokines in macrophages (Figure S2A,B, Supporting Information), O‐Mit was selected for further in vivo study. B6 mice were intranasally administered of LPS (50 µg mice^−1^). Six hours later, the ALI mice were gavage‐given of O‐Mit (1 mg mice^−1^) and a variety of tests were conducted on the ALI mice (**Figure** **1**A). We found that the rectal temperature of LPS‐treated mice (ALI) rapidly dropped from 36.6 ± 0.24 to 31.6 ± 0.57 °C (*p* = 0.008) at 6 h. In the group of ALI mice that were gavage‐given O‐Mit, their rectal temperature increased rapidly and reached the normal temperature of 35.80 ± 0.58 °C at 12 h, which was significantly higher than the rectal temperature of 33.26 ± 0.40 °C (*p* = 0.006) in the ALI mice without O‐Mit treatment. (Figure 1B). LPS induces ALI in mice, making the lung wet/dry weight ratio a suitable indicator for assessing pulmonary edema.^[^ ^33^, ^34^ ^]^ Experimental results demonstrated an increase in the lung wet/dry weight ratio in ALI mice. However, gavage administration of O‐Mit reduced the wet/dry weight ratio, reversing lung tissue inflammation in the ALI mice (Figure 1C). These data agreed with the inhibition in the induction of an array of inflammatory cytokines. Cytokine array analysis of mouse lung tissue revealed that the levels of cytokines IL‐1β, IFN‐γ, and IL‐6 were elevated, while the anti‐inflammatory cytokine IL‐10 was decreased in the ALI mice. Whereas, O‐Mit inhibited LPS‐induced elevation of IL‐1β, IFN‐γ, IL‐6, and alleviation of IL‐10 (Figure 1D). These data were consistent with the enzyme‐linked immunosorbent assay (ELISA) analysis of cytokines in mouse BALF (Figure 1E) and serum samples (Figure 1F). In BALF, ELISA data indicated that O‐Mit significantly inhibited LPS‐induced induction of IL‐1β (*p* = 0.041), IFN‐γ (*p *= 0.0003), IL‐6 (*p* = 0.0012), and alleviation of IL‐10 (*p* = 0.003) (Figure 1E). In serum, O‐Mit significantly attenuate LPS‐mediated activation of IL‐1β (*p *= 0.008), IFN‐γ (*p* = 0.0006), IL‐6 (*p* = 0.0002), and repression of IL‐10 (*p* = 0.007) (Figure 1F). Hematoxylin and eosin (H&E) staining of lung tissue sections demonstrated O‐Mit reversed the severe inflammation as judged by infiltration of inflammatory cells and thickening of the alveolar wall in the lung tissue of ALI mice (Figure 1G).

To determine if inflammatory cytokine genes were being altered at a transcriptional level, total RNA was isolated from lung tissues of the ALI mouse subjected to the different treatments. The q‐PCR array analysis suggested that the gavage‐given O‐Mit had decreased expression levels of the pro‐inflammatory cytokine genes IL‐6, IL‐1α, IL‐1β, CXCL1/2, and IFN‐γ, while the O‐Mit‐treated mice had an increased expression of the anti‐inflammatory genes IL‐27p28 and thrombopoietin when compared to the cytokine genes in the lung tissues of ALI mice^[^ ^35^ ^]^ (Figure 1H). To further verify the q‐PCR array results, individual q‐PCR was used to assess the expression of cytokine genes in the lung tissue of the ALI mouse receiving different treatments. Compared with the lung tissues of ALI mice, gavage‐given O‐Mit reduced the expression of pro‐inflammatory IL‐6 (*p* = 0.0003), IFN‐γ (*p* = 0.0004), IL‐1α (*p* = 0.0007), while increasing the expression of anti‐inflammatory IL‐27p28 (*p* = 0.004) in the ALI mice (Figure 1I). Monocytes such as macrophages in lung alveolar and interstitial spaces are the main target cells of LPS and are the first to produce inflammatory cytokines.^[^ ^36^, ^37^, ^38^ ^]^ Based on this fact we isolated monocytes from the lungs of ALI mice for proinflammatory factor analysis. Fluorescence‐activated cell sorting (FACS) analysis suggested that O‐Mit significantly inhibits LPS‐induced IFN‐γ (Figure 1J) and tumor necrosis factor alpha (TNF‐α) (Figure 1K) production in lung monocytes. Collectively, the data suggest that oral administration of O‐Mit inhibits LPS‐induced ALI in mice.

### 2.2 Lung Macrophages Take Up O‐Mit in a Phosphatidic Acid (PA) Dependent Manner

**Figure 2:**
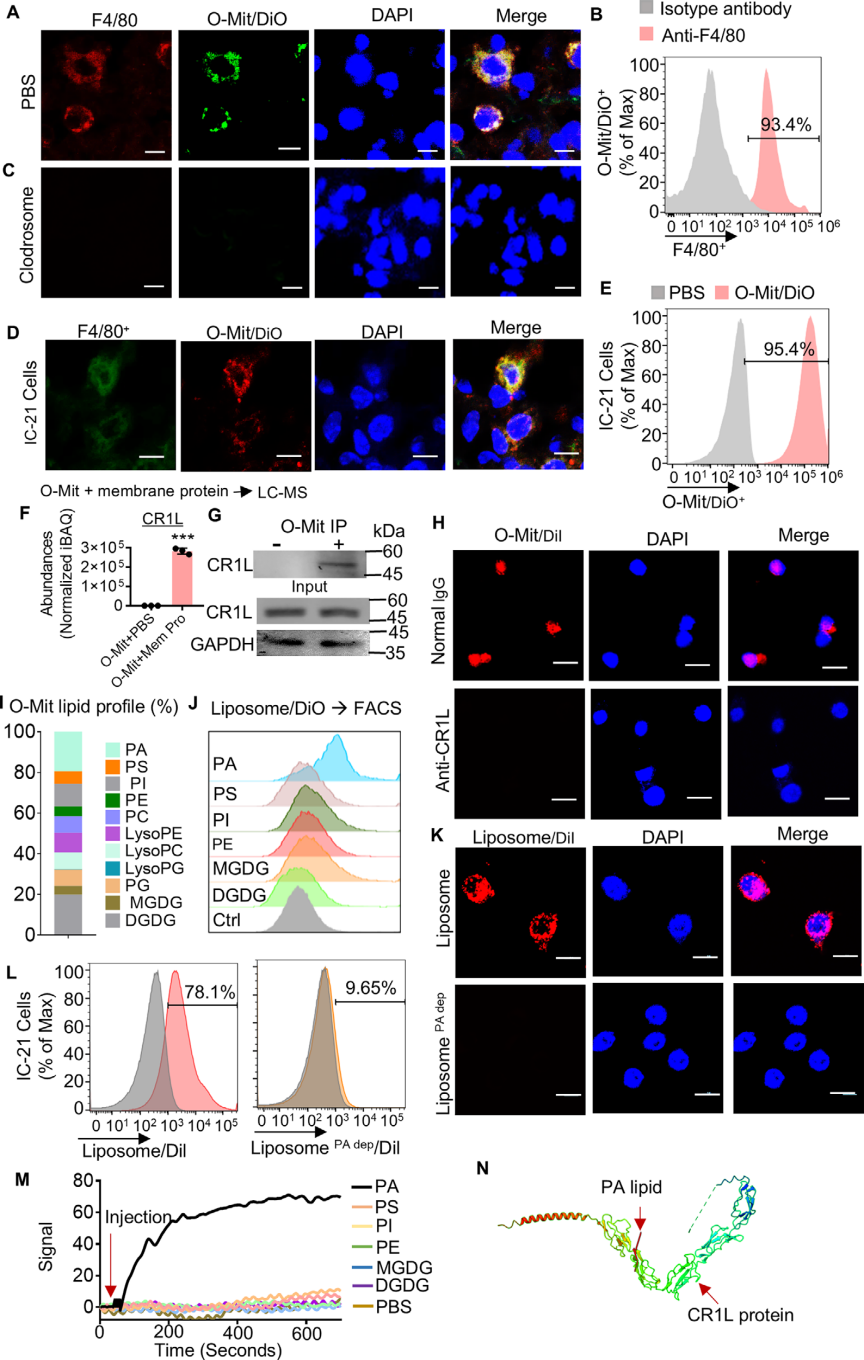
O‐Mit taken up by macrophages is mediated by the interaction between PA and CR1L. A) ALI mice were administered with fluorescence dye‐labeled O‐Mit via oral gavage (1 mg each^−1^, *n* = 5). Two hours post gavage, the confocal microscopy showed colocalization of O‐Mit/DiO with F4/80^+^ cells in lung tissue (400× magnification, scale bar 200 µm). B) FACS analysis was used to quantify the uptake of DiO‐labeled O‐Mit by F4/80^+^ cells in the lung tissue of ALI mice. C) ALI mice were intraperitoneally injected with clodronate liposomes to deplete macrophages at a dose of 80 mg kg^−1^ body weight. Three days later, 50 µg of LPS was administered intranasally, followed by oral gavage of 1 mg of O‐Mit/DiO. Lung tissues were then collected, frozen‐sectioned, and observed using confocal microscopy (400× magnification, scale bar 200 µm). No F4/80^+^ cells or O‐Mit/DiO were detected in the lung tissue of the mice. D) Macrophage IC‐21 cells were co‐incubated with DiO‐labeled O‐Mit. After removing the supernatant, the macrophages were washed with PBS and DAPI to stain nuclei. The uptake of O‐Mit by macrophages was observed using confocal microscopy. (400× magnification, scale bar, 200 µm). E) FACS was used to analyze the uptake of DiO‐labeled O‐Mit by macrophages. F) O‐Mit was incubated with macrophage membrane proteins, then centrifuged to collect O‐Mit. The O‐Mit‐interacting with the macrophage membrane protein was analyzed using LC‐MS. O‐Mit incubated with PBS used as negative control (***, *p* < 0.0001, two‐way *t‐*test). G) O‐Mit was incubated with macrophage membrane proteins and centrifuged to collect O‐Mit. After lysis with CTAB lysis buffer, co‐immunoprecipitation (co‐IP) was used to detect the binding of O‐Mit to the macrophage membrane protein CR1L. H) anti‐CR1L antibody (2 µg mL^−1^) was incubated with macrophage IC‐21 cells, then DiI‐labeled O‐Mit was co‐incubated with CR1L antibody‐treated macrophages. The macrophages were stained with DAPI, and the uptake of O‐Mit by macrophages was observed using a confocal microscope. (400× magnification, scale bars, 200 µm). I) Chloroform was used to extract lipids from the purified O‐Mit, and the lipid profile of O‐Mit was detected using LC‐MS. J) DiO‐labeled liposomes prepared with different lipids were incubated with macrophages. After removing the supernatant, the DiO‐labeled liposomes taken up by macrophages were analyzed using FACS. K) Thin‐layer chromatography (TLC) was used to separate O‐Mit lipids. The separated O‐Mit lipids were used to prepare liposomes with PA‐depleted lipids (liposome ^PA dep^) and liposomes without PA depletion using a liposome preparation instrument. The liposomes were labeled with DiI and incubated with macrophages. Uptake of liposomes by macrophages was then observed using confocal microscopy. (400× magnification, scale bars, 200 µm). L) FACS was used to analyze the uptake of Dil‐labeled liposome with or without PA by macrophages. M) The hydrophobic chip was placed in the Open SPR instrument. Immobilize different lipids were run over the hydrophobic chip, CR1L protein was injected onto the hydrophobic chip, and the signal changes observed on the Open SPR. N) The 3D structures of AP and CR1L proteins were simulated for binding using the online platform HDOCK SERVER. individual groups were analyzed via two‐way ANOVA. Data are representative of three independent experiments (error bars, SD). ** *p* < 0.01.

Based on published data,^[^ ^39^ ^]^ nanoparticles from edible plants can be taken up by macrophages.^[^ ^40^ ^]^ To determine whether lung macrophages in ALI mice take up the O‐Mit, ALI mice were gavage‐given DiO‐labeled O‐Mit. Two hours after the gavage, ALI mice were euthanized, frozen sections were prepared from the lungs of the mice, nuclei were stained with DAPI, and macrophages were stained with anti‐F4/80 antibody. Confocal microscopy results showed that DiO‐labeled O‐Mit co‐localized with anti‐F4/80 positive macrophages in lung tissue, suggesting that O‐Mit is taken up by macrophages in the lungs of ALI mice (**Figure** **2**A). To confirm the confocal result, Percoll gradient‐isolated lung leukocytes^[^ ^41^ ^]^ were FACS analyzed. The results indicated that 93.4% of O‐Mit/DiO^+^ recipient cells are F4/80^+^ lung macrophages. (Figure 2B). To further determine whether lung macrophages are the predominant cells that take up O‐Mit, B6 mouse macrophages were depleted by intraperitoneal injection of Clodronate Liposome^[^ ^40^, ^42^ ^]^ in ALI mice. The mice were gavage‐given DiO‐labeled O‐Mit. Confocal image results indicated that depletion of macrophages results in there being no DiO‐labeled O‐Mit detected in the sectioned lung tissue (Figure 2C). To further verify this result, the B6‐derived macrophage cell line IC‐21^[^ ^43^, ^44^ ^]^ was incubated with DiO‐labeled O‐Mit. The cells were washed with PBS and stained with anti‐F4/80 antibody. Confocal microscopy results showed that a large amount of O‐Mit was taken up by the macrophage cells (Figure 2D). The confocal results were also supported by FACS, where 95.4% of the O‐Mit/DiO was taken up by macrophages (Figure 2E). The influence of LPS on the phagocytic activity of macrophages varies depending on the cell type. We conducted a phagocytic assay using the Vybrant Phagocytosis Assay Kit. The results suggested that LPS (10 µg mL^−1^) enhances macrophage phagocytosis (Figure S7A, Supporting Information), which partially explains why O‐Mit preferentially traffics to the lungs of ALI mice. O‐Mit up take is likely lung macrophage specific since O‐Mit/DiO signals do not co‐localize with epithelial cell adhesion molecule (EpCAM), a marker of epithelial cells in the lungs (Figure S7B, Supporting Information). Although gut epithelial cell mitochondria (E‐Mit) can also traffic to the lungs (Figure S5E, Supporting Information) and be taken up by macrophages (Figure S7C, Supporting Information), unlike O‐Mit, E‐Mit showed little impact on LPS‐induced inflammation (Figure S7D, Supporting Information).

Next, to identify which molecule on the macrophage‐mediated O‐Mit entry, the macrophage IC‐21 cell plasma membrane proteins were isolated using sucrose gradient ultracentrifugation^[^ ^45^ ^]^ and incubated with O‐Mit. The O‐Mit‐interacting complexes were collected and identified using liquid chromatography‐mass spectrometry (LC‐MS) analysis. The results indicated that the complement C3b/C4b receptor 1 like (CR1L) protein of the macrophage membrane was potentially interacting with the O‐Mit (Figure 2F). This LC‐MS result of the CR1L protein in the O‐Mit‐interacting membrane protein complex was subsequently confirmed by western blot (WB) (Figure 2G). To further determine whether macrophage CR1L plays a causative role in the uptake of the O‐Mit, macrophage IC‐21 cells were pretreated with anti‐CR1L antibody to block the potential interaction of CR1L with O‐Mit. We found that the uptake of O‐Mit in IC‐21 cells was abolished by pre‐incubation of anti‐CR1L antibody with O‐Mit (Figure 2H). These results demonstrate that the uptake of O‐Mit by macrophages is primarily mediated by the interaction of macrophage membrane protein CR1L with O‐Mit.

Lipids from edible plant nanoparticles have been shown to play a role in the uptake of plant nanoparticles by macrophages.^[^ ^46^ ^]^ Next, we tested whether and which lipid of O‐Mit interacts with CR1L. We extracted the lipids from O‐Mit and analyzed their composition using LC‐MS. The results showed that the O‐Mit predominately contains the lipids PA, PS, PI, PE, MGDG, and DGDG (Figure 2I). Using TLC, the extracted O‐Mit lipids were separated, and the different types of lipids were collected. The individual lipids extracted from TLC were processed into liposomes using a nanogenerator (NanoGenerator Flex‐S System). The FACS analysis indicated that macrophages take up more liposomes prepared from PA lipids than those made from the other lipids (Figure 2J). DiI‐labeled liposomes with PA or PA depletion in TLC were used to determine whether PA is essential for uptake by macrophages. Confocal microscopy results revealed that liposomes with PA depletion were not taken up by macrophages (Figure 2K). FACS analysis also suggested that PA depletion caused a decrease in liposome uptake by macrophages from 78.1% to 9.65% (Figure 2L). Next, surface plasmon resonance (SPR) was used to determine the direct interaction between PA and CR1L. We installed a hydrophobic chip on an OpenSPR and injected prepared lipid solutions onto the hydrophobic chip. The CR1L protein solution was introduced onto the chip coated with different types of lipids, and binding signals were monitored. When the CR1L protein solution was injected into the chip coated with PA lipid, the OpenSPR showed a strong signal, confirming that the CR1L protein interacts directly with the PA lipid (Figure 2M). Finally, to investigate the interaction between PA and the CR1L protein, we performed computational 3D structure modeling predictions. The 3D structures of PA and the CR1L protein were submitted to the HDOCK SERVER online platform and analyzed using Chimera 1.9 software. Based on the top three best‐fitting models, the docking score ranged from −216.6 to −208.15, and the ligand RMSD (Å) ranged from 34.18 to 56.36. We speculate that the binding PA to the CR1L protein plays a crucial role in macrophage uptake of O‐Mit (Figure 2N).

### 2.3 Fusion of O‐Mit and Macrophage Mitochondria (M‐Mit)

**Figure 3:**
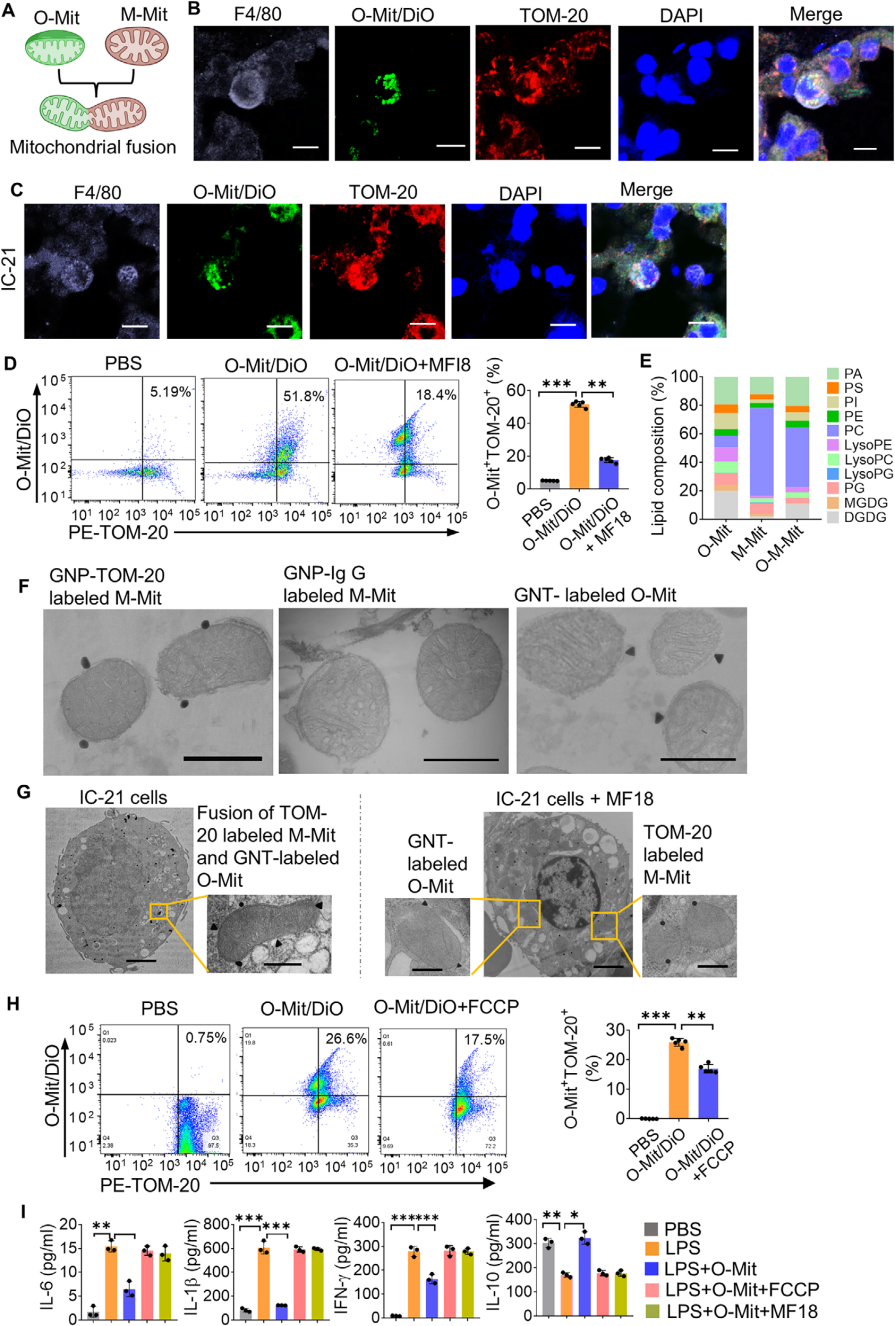
Fusion of O‐Mit and macrophage mitochondria (M‐Mit). A) Schematic illustration of the fusion between O‐Mit and macrophage mitochondria (M‐Mit) after being taken up by macrophages. B) ALI mice were gavaged with DiO‐labeled O‐Mit. Lung tissue from ALI mice was frozen‐sectioned and stained with anti‐F4/80 to label macrophages, anti‐TOM‐20 to label M‐Mit, and DAPI to label nuclei. Confocal microscopy revealed that O‐Mit was taken up by macrophages in the lung tissue of ALI mice and fused with M‐Mit. (400× magnification, scale bars, 200 µm). C) DiO‐labeled O‐Mit was incubated with macrophages. Anti‐F4/80 was used to label macrophages, anti‐TOM‐20 to label M‐Mit, and DAPI to label nuclei. Confocal microscopy revealed that O‐Mit was taken up by macrophages and fused with M‐Mit. (400× magnification, scale bars, 200 µm). D) DiO‐labeled O‐Mit was incubated with macrophages 1h, and mitochondria were then isolated from the macrophages. PE‐TOM‐20 antibody was used to label the M‐Mit. FACS analysis showed that O‐Mit interacted with M‐Mit and this interaction was abolished by mitochondrial fusion inhibitor MFI8 (left panel). Quantification of O‐Mit/DiO^+^TOM20^+^ (right panel). E) After incubating O‐Mit with macrophages, M‐Mit were isolated, and O‐Mit/DiO^+^ M‐Mit (O‐M‐Mit) were sorted by FACS, followed by lipid compositions analysis using LC‐MS. F) Gold‐sphere nanoparticle (GNP) labeled M‐Mit (left), GNP IgG labeled M‐Mit (middle), and gold‐triangle nanoparticle (GNT) labeled O‐Mit (right) visualized by TEM. Scale bar, 800 nm. G) In vitro, GNT labeled O‐Mit incubated with macrophages at 37 °C for 1 h. After washing, M‐Mit is labeled with anti‐Tom20 and GNP‐protein A beads. A representative TEM of macrophage cells (left, Scale bar, 2 µm) and magnification of mitochondria in macrophages (right, Scale bars, 800 nm). H) FACS analysis of O‐Mit activity and its effect on mitochondrial fusion in macrophages (left panel). Quantification of O‐Mit/DiO^+^TOM20^+^ (right panel). I) O‐Mit was unable to inhibit LPS‐induced cytokine secretion in macrophages following treatment with FCCP or MF18. Differences between percentages were analyzed with a chi‐square test. Differences between individual groups were analyzed via two‐way ANOVA. Data are representative of three independent experiments (error bars, SD). * *p* < 0.05, ** *p* < 0.01, *** *p* < 0.001.

Mitochondrial fusion and fission take place in both plants and mammalians. In each case, a dynamic balance of fusion and fission is maintained which is crucial for preserving normal morphology, distribution, and function of mitochondria. However, whether mitochondrial fusion occurs across kingdoms has never been investigated. We hypothesize that once O‐Mit enters the macrophages, O‐Mit may have effects on M‐Mit activity through fusing with M‐Mit (**Figure** **3**A). To test this hypothesis, we administered DiO‐labeled O‐Mit to ALI mice via gavage. Two hours after being gavage, the mice lung tissue sections were prepared. Macrophages were stained with anti‐F4/80 antibody, and their mitochondria were stained with anti‐TOM‐20 antibody. Confocal microscopy images suggested that the DiO‐labeled O‐Mit colocalizes with the M‐Mit marker F4/80 and mitochondrial marker TOM‐20 (Figure 3B). In vitro confocal imaging results (Figure 3C) also agreed with the in vivo confocal results. FACS analysis further showed that 51.8% of the O‐Mit was interacting with the M‐Mit (O‐Mit/DiO^+^TOM‐20^+^). Mitochondria fusion inhibitor MFI8 treatment completely inhibited the interaction (Figure 3D), indicating that O‐Mit and M‐Mit fused. Additionally, the results of LC‐MS analysis of the extracted M‐Mit lipids demonstrated that the ratios of mitochondria lipid composition were altered after incubation with O‐Mit. Notably, the plant‐specific lipid DGDG was detected in the M‐Mit following co‐incubation with O‐Mit (Figure 3E).

To obtain direct evidence that O‐Mit fuses with M‐Mit we conducted TEM. We labelled the M‐Mit and O‐Mit with distinctive shape spherical (GNP) and triangular (GNT) gold‐nanoparticles, respectively (Figure 3F). Next, macrophage IC‐21 cells were incubated with the GNT‐labeled O‐Mit and fixed in 4% paraformaldehyde buffer solution. The macrophages were then embedded in resin and sectioned into ultrathin slices for TEM. The embedded macrophages were sectioned and stained with GNP‐labeled anti‐TOM‐20 antibody and normal lgG was used as a negative control (Figure 3F). The TEM images revealed the presence of GNT‐labeled O‐Mit within macrophages, co‐localized with the TOM20/GNP‐positive mitochondria (Figure 3G). Mitochondria specific fusion inhibitor MF18 attenuated the colocalization of GNP (O‐Mit) and GNT (TOM‐20) (Figure 3G). These TEM images provide direct evidence that macrophages take up O‐Mit and the O‐Mit fuses with the M‐Mit. Mitochondrial fusion is a complicated process and maintaining the integrity of mitochondrial membrane potential is necessary.^[^ ^47^ ^]^ Our data identified that O‐Mit fused with M‐Mit is dependent on the activity of the O‐Mit membrane potential (Figure 3H). O‐Mit membrane potential disrupted by the mitochondrial uncoupler FCCP abolished the O‐Mit fusion with M‐Mit (Figure 3H) and the capacity for anti‐inflammatory activity (Figure 3I).

### 2.4 O‐Mit Rescue M‐Mit Dysfunction in the Lungs of ALI Mice

Mitochondria are often denoted as the powerhouses of the cell. Besides ATP production, mitochondria are involved in other important processes such as regulating cellular metabolism. Dysfunctional mitochondria are linked to a variety of diseases, including certain types of lung inflammation‐related diseases. Mitochondrial‐induced inflammation is significant in a variety of diseases, including ALI.^[^ ^14^, ^48^, ^49^ ^]^ When becoming dysfunctional, mitochondria produce excess reactive oxygen species (ROS). Excess mitochondrial superoxide (a specific type of ROS) plays a significant role in regulating various mitochondrial functions, including the levels of mitochondrial GTP, adenosine triphosphate (ATP), and GSSG.^[^ ^50^, ^51^, ^52^ ^]^ GTP, ATP, and GSSG each play distinct yet interconnected roles in mitochondrial function. For example, maintaining a proper balance of glutathione (GSH) and GSSG is crucial for regulating inflammation and preventing excessive cytokine production.

**Figure 4:**
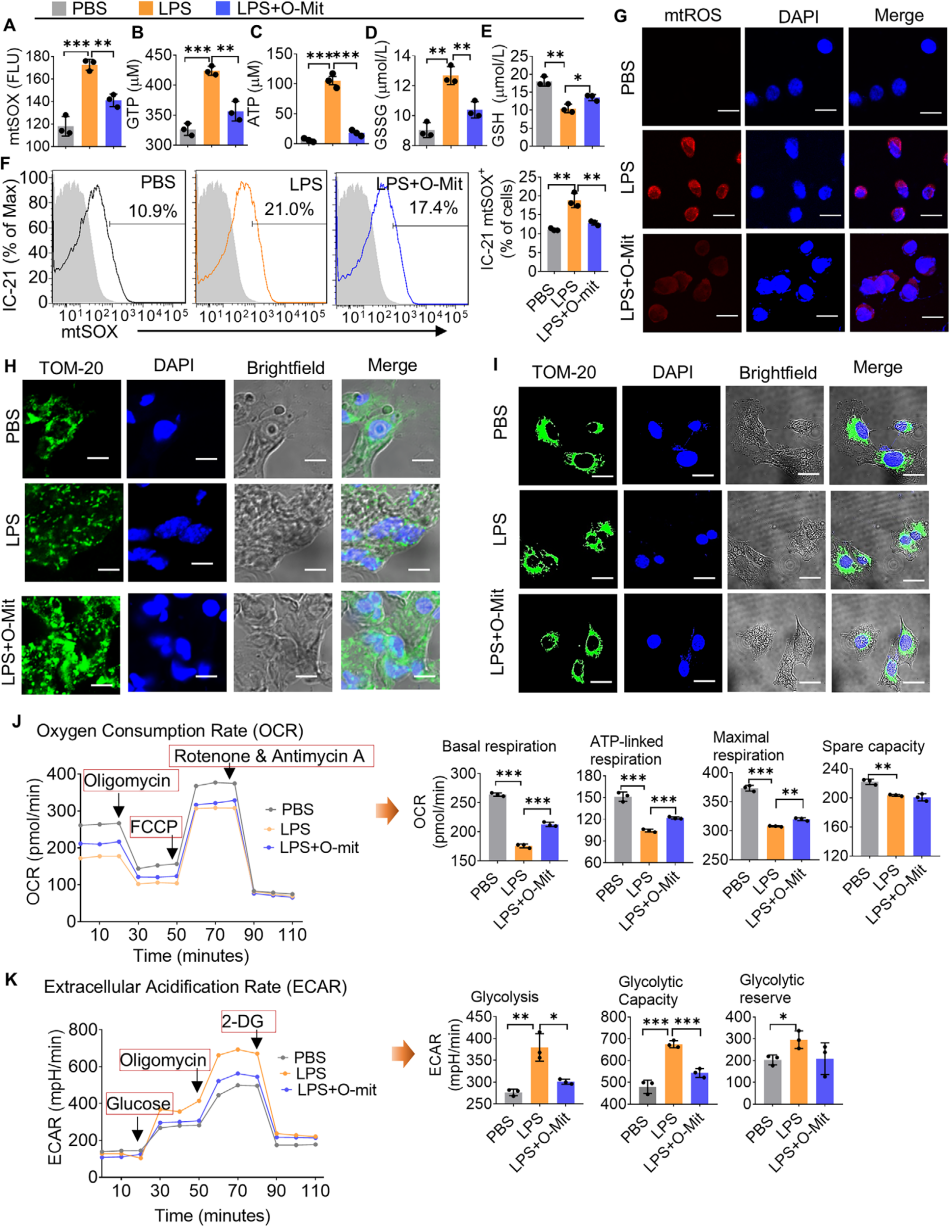
O‐Mit regulates the mitochondrial activities of macrophages in the lung. A–E) Macrophage IC‐21 was incubated with LPS (1 µg mL^−1^) for 6 h followed by treating with O‐Mit (1 µg mL^−1^). Macrophage mitochondrial activities were assessed by measuring A) mtSOX, B) GTP, C) ATP, D) GSSG, and E) GSH. F) Macrophages were incubated with LPS and after being washed with PBS, the macrophages were treated with O‐Mit. FACS analysis was used to assess macrophage mitochondrial superoxide (mtSOX). The results indicated that O‐Mit reduced LPS‐induced mtSOX in macrophages. G) Macrophages were incubated with LPS, and after washing with PBS, the macrophages were treated with or without O‐Mit. Confocal microscopy observation revealed that O‐Mit reduced LPS‐induced ROS in macrophages. (400× magnification, scale bars, 200 µm). H,I) Confocal microscopy was used to observe the morphology of mitochondria. In the lung tissue of ALI mice (H) and in macrophages (I), LPS induced mitochondrial fragmentation. After treatment with O‐Mit, the mitochondria in the cells were restored to normal mitochondrial morphology. (400× magnification, scale bars, 200 µm). J,K) Macrophages were treated with LPS, after washing with PBS, the O‐Mit was incubated with macrophages. Oxygen consumption rate (OCR) (J) and extracellular acidification rate (ECAR) (K) were measured using Seahorse analysis. The results showed that O‐Mit reduced LPS‐induced mitochondrial ECAR and increased the mitochondrial OCR in macrophages. Differences between percentages were analyzed with a chi‐square test. Differences between individual groups were analyzed via two‐way ANOVA. Data are representative of three independent experiments (error bars, SD). * *p* < 0.05, ** *p* < 0.01, *** *p* < 0.001.

We hypothesized that the altering of mitochondrial activity in macrophages is the primary mechanism by which O‐Mit reduces lung inflammation in ALI mice. To test this hypothesis, macrophage IC‐21 cells were exposed to LPS followed by incubation with O‐Mit. The analysis of mitochondrial activities in IC‐21 cells indicated that LPS induces mitochondrial superoxide (mtSOX) production (**Figure** **4**A), mitochondrial GTP, ATP, and GSSG (Figure 4B–D), while reducing the level of GSH (Figure 4E). In contrast, O‐Mit treatment dramatically inhibits the LPS‐mediated induction of mtSOX, GTP, ATP, and GSSG (Figure 4A–D), as well as LPS‐mediated reduction of GSH (Figure 4E). The result that LPS‐induced mtSOX production was inhibited by O‐Mit was further confirmed by FACS analysis (Figure 4F) and confocal microscopy images (Figure 4G). Collectively, these experimental results suggest that the O‐Mit inhibits the LPS‐induced oxidative stress response in M‐Mit.

Changes in the shape of mitochondria can affect their function. Mitochondria constantly undergo dynamic processes known as fusion (joining together) and fission (splitting apart), which allow them to adapt to the needs of the cell. We fixed the ALI mouse lung tissue and stained the nuclei with DAPI and the mitochondria with anti‐TOM‐20 antibody. Confocal examination results showed that in the PBS‐treated control group of B6 mice, the mitochondria in lung tissue cells displayed a filamentous or sheet‐like distribution. However, the mitochondria exhibited a punctate and dispersed distribution in ALI mice. In the mice that were gavage‐given O‐Mit, most of the mitochondria in the lung tissue appeared like the control group, with a filamentous or sheet‐like distribution, with only a few displaying a punctate and dispersed pattern (Figure 4H). The results generated from an in vivo study corroborated the data generated from in vitro O‐Mit‐treated macrophage IC‐21 cells (Figure 4I). These experimental results indicate that O‐Mit treatment prevents the mitochondria shape changes induced by LPS stimulation.

Changes in the shape of mitochondria can affect their function.^[^ ^53^ ^]^ Using a Seahorse XF analyzer, the oxygen consumption rate (OCR) and the extracellular acidification rate (ECAR) in macrophage IC‐21 cells were assessed to evaluate their mitochondrial function. The results showed that under LPS stimulation, the inflammatory activation of macrophages led to a decrease in mitochondrial OCR. But O‐Mit alleviated the reduction of the mitochondrial OCR (Figure 4J, left panel) such as basal respiration, ATP‐linked respiration, and maximal respiration (Figure 4J, right panel). We also found that O‐Mit inhibits the effect of LPS‐mediated induction in ECAR (Figure 4K, left panel) including glycolysis, glycolytic capacity, and glycolytic reserve (Figure 4K, right panel).

### 2.5 O‐Mit Methyl 3,4‐dihydroxybenzoate (MDHB) Inhibits LPS‐Induced Lung Inflammation

As shown in Figure 4 and O‐Mit can inhibit LPS‐mediated induction of ROS, which is capable of activating the production of proinflammatory cytokines such as IL‐1β, IFN‐γ, and IL‐6. Next, we sought to identify which O‐Mit factor(s) inhibit the LPS‐mediated induction of inflammatory cytokines. O‐Mit contains multiple factors such as protein, nucleic acids, and small molecules.^[^ ^54^, ^55^, ^56^ ^]^ To identify which O‐Mit content is responsible for the anti‐inflammatory effects, we treated the O‐Mit with proteases and RNase, to remove the proteins and RNAs of O‐Mit (Figures S8A, Supporting Information). It is well‐established that mitochondrial proteins are essential for maintaining membrane potential (Δψm) and enabling mitochondrial fusion.^[^ ^57^ ^]^ To protect O‐Mit from protease‐induced damage and gut environmental stress, we stored isolated O‐Mit in a modified maintain buffer^[^ ^23^ ^]^ prior to treatment. This buffer includes disodium hydrogen phosphate (Na_2_HPO_4_),^[^ ^58^ ^]^ sodium pyruvate,^[^ ^59^ ^]^ EGTA, and mitochondrial stabilizers such as Ginkgo biloba extract Egb761. Phosphate and pyruvate support mitochondrial respiration and help preserve membrane potential,^[^ ^58^, ^59^ ^]^ while EGTA chelates calcium ions that can trigger depolarization.^[^ ^60^ ^]^ Egb761 has been widely used to stabilize mitochondrial structure and function.^[^ ^61^ ^]^ Analysis of membrane potential suggested that O‐Mit preserved in the modified maintenance buffer (MB) retained 88.6 ± 6.3% of Δψm after protease treatment (*p* = 0.22), compared to only 52.1 ± 5.5% (*p* = 0.0005) of Δψm retained in regular MB (Figure S8B, Supporting Information). As we expect, RNase has no impact on the membrane potential of O‐Mit.

**Figure 5:**
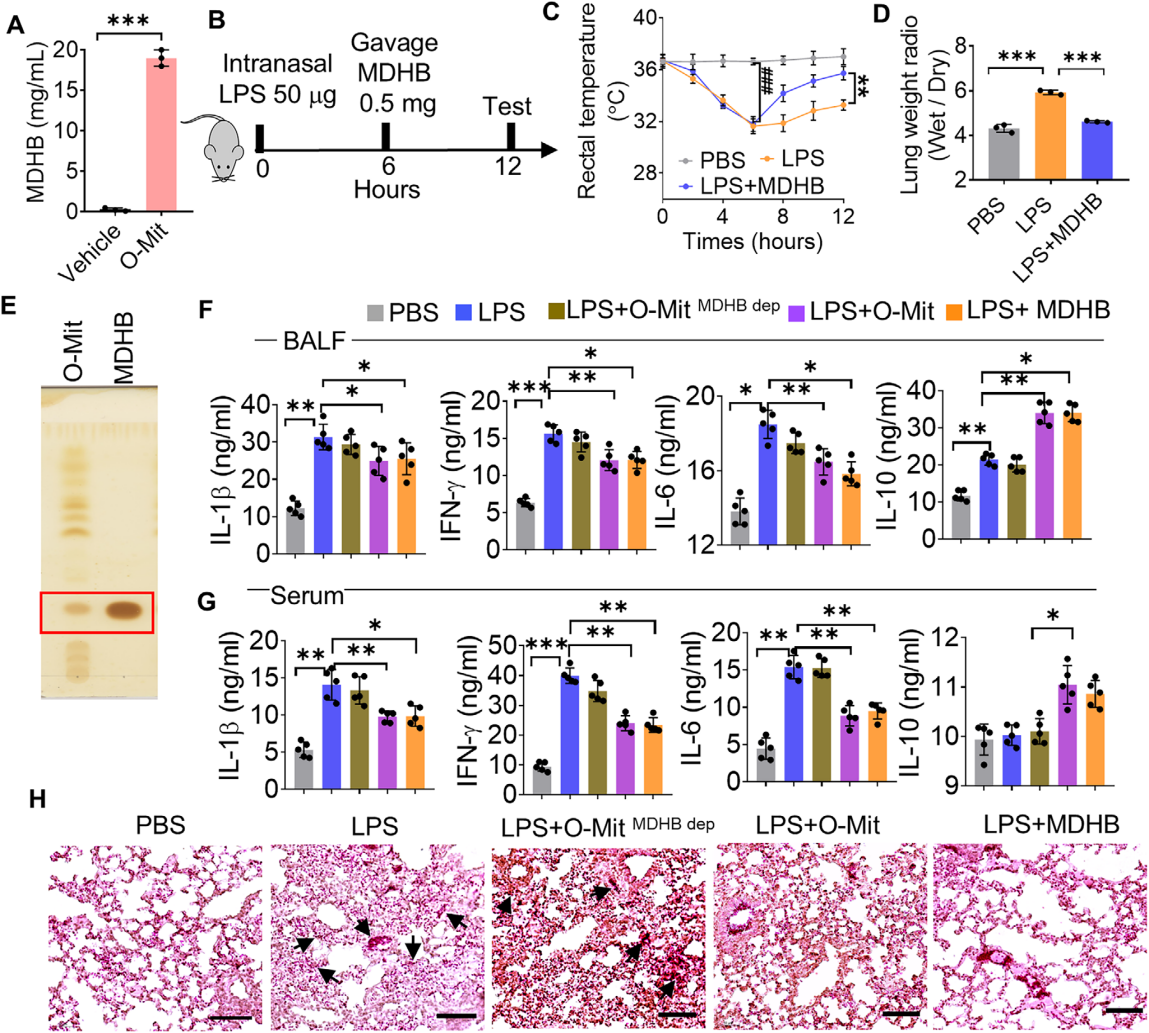
Methyl 3,4‐dihydroxybenzoate (MDHB) in O‐Mit inhibits LPS‐induce ALI mice. A) O‐Mit was incubated with macrophage membrane proteins, then centrifuged to precipitate O‐Mit, which was lysed using CTAB lysis buffer. LC‐MS analysis of small molecules in the O‐Mit CTAB lysate revealed an enrichment of MDHB. B) Schematic illustration of the experiment: C57BL/6J mice were intranasally administered 50 µg mice^−1^ of LPS. After 6 h the mice were gavaged with 0.5 mg mice^−1^ of MDHB. Six hours after the gavage, cytokines in the mice were assessed. C) Rectal temperatures of B6 mice were recorded every 2 h after intranasal LPS. At 6 h, a significant difference was observed between the LPS and PBS groups (^##^, *p *< 0.001). MDHB (0.5 mg mouse^−1^, *n* = 5) was given orally at 6 h. After 6 h, MDHB‐treated mice showed a significant temperature difference versus the LPS group (**, *p* < 0.01). D) The lung wet/dry weight ratio was used to assess pulmonary edema in mice from different treatment groups. E) Thin‐layer chromatography (TLC) was used to separate the small molecules in the O‐Mit lysate. The red box indicates that the MDHB band in the O‐Mit lysate according to the migration of the MDHB standard. F,G) ALI mice were gavaged with O‐Mit lysate, MDHB‐removed O‐Mit lysate (O‐Mit ^MDHB dep^), and MDHB. After 6 h, cytokines IL‐1β, IFN‐γ, IL‐6, and IL‐10 in the bronchoalveolar lavage fluid (BALF) (F) and serum (G) of ALI mice were measured by ELISA. The results indicated that ALI mice gavaged with O‐Mit lysate and MDHB reduced pro‐inflammatory cytokines IL‐1β, IFN‐γ, and IL‐6, and increased the anti‐inflammatory cytokine IL‐10. ALI mice gavaged with O‐Mit ^MDHB dep^ had cytokine expressions that were similar to that of the ALI mice. H) Hematoxylin and eosin (H&E) stained images of lung tissue from ALI mice showed that lung inflammation was significantly reduced after gavage with O‐Mit lysate and MDHB. However, when ALI mice were gavaged with O‐Mit ^MDHB dep^ lung inflammation was observed to be similar to that the ALI mice. (Original magnification X20. Bar scale, 100 µm). The arrows indicate the infiltration of inflammatory cells in the alveolar cavity and interstitial area, as well as the thickening of the alveolar wall. Differences between individual groups were analyzed via two‐way ANOVA. Data are representative of three independent experiments (error bars, SD). * *p* < 0.05, ** *p *< 0.01, *** *p* < 0.001.

The protein or RNAs free O‐Mit were then gavage‐given to ALI mice. H&E staining of lung tissue (Figure S8C, Supporting Information), as well as cytokines analysis in BALF (Figure S8D, Supporting Information) and serum (Figure S8E, Supporting Information) showed that O‐Mit inhibits LPS‐induced inflammation, and neither protease nor nuclease influences the action of O‐Mit (Figure S8C–E, Supporting Information), suggesting that O‐Mit factors other than protein and nucleic acids such as small molecules play the crucial role of anti‐inflammation response. Our metabolomics analysis of O‐Mit using LC‐MS indicated that among O‐Mit metabolic products (Table S1, Supporting Information), methyl 3,4‐dihydroxybenzoate (MDHB) was enriched (**Figure** **5**A). Given MDHB can inhibit the LPS‐induced inflammation in the lung and osteolysis,^[^ ^62^, ^63^ ^]^ we then tested if MDHB contributes to O‐Mit‐mediated anti‐inflammation in ALI mice. ALI mice were gavage‐given 0.5 mg mice^−1^ MDHB (Figure 5B). We found that ALI mice treated with MDHB showed a dramatic recovery from the LPS‐induced drop in rectal temperature, which was more effective than in ALI mice without MDHB treatment (35.7 ± 0.52 vs 33.26 ± 0.40 °C, *p* = 0.0005) (Figure 5C). The lung wet/dry weight ratio result demonstrated an increase in the lung wet/dry weight ratio in ALI mice. However, after gavage‐giving mice MDHB, the lung wet/dry weight ratio decreased in the ALI mice (Figure 5D). To further test the causal role of MDHB in anti‐inflammation, MDHB was depleted from O‐Mit (O‐Mit^MDHB dep^) using TLC^[^ ^64^ ^]^ (Figure 5E). Then, ALI mice were gavage‐given O‐Mit, O‐Mit^MDHB dep^, or MDHB (0.5 mg mice^−1^). The ELISA analysis showed that compared to the O‐Mit or MDHB‐treated group, gavage administration of O‐Mit^MDHB dep^ had no significant effect on the LPS‐mediated induction of IL‐1β, IFN‐γ, IL‐6, or the reduction of IL‐10 in the BALF (Figure 5F) and serum (Figure 5G) of the ALI mice. H&E stained images showed that the lung tissues of ALI mice and mice gavage‐given O‐Mit^MDHB dep^ exhibited more intense inflammation compared to the O‐Mit lysate and MDHB‐treated groups. The cytokine results were consistent with the H&E staining results (Figure 5H).

### 2.6 O‐Mit MDHB Rescues M‐Mit Dysfunction

**Figure 6:**
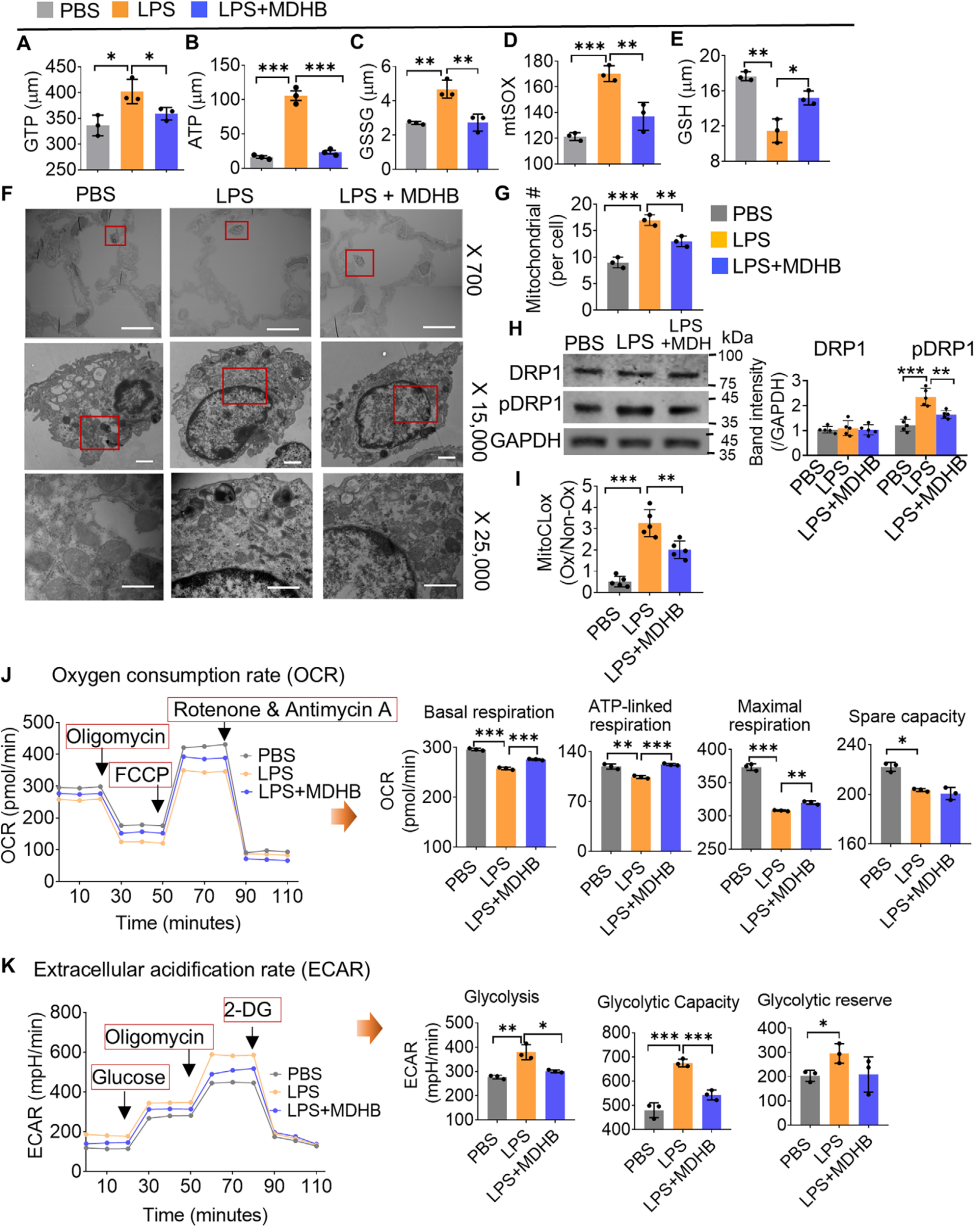
MDHB rescues LPS‐induced macrophage mitochondrial dysfunction. A–E) Macrophage IC‐21 cells were incubated with LPS (1 µg mL^−1^) for 6 h followed by treating with MDHB (0.5 µg mL^−1^). Macrophage mitochondrial activities were assessed by measuring A) GTP, B) ATP, C) GSSG, D) mtSOX, and E) GSH. F) ALI mice were gavage‐given MDHB (0.5 mg mice^−1^) and lung tissue was collected 6 h later. TEM was used to measure the mitochondrial substructures and number in macrophages within the lung tissue of mice (top panel, ×700, Scale bar, 10 µm; middle panel, ×15 000, Scale bar, 2 µm; bottom panel, ×25 000, Scale bar, 600 nm). G) The number of mitochondria in macrophages in ALI mouse lung tissue with or without MDHB gavage observed by TEM. H) Western blot analysis of the mitochondrial fission protein DRP1 and phosphorated DRP1 (pDRP1) in the lung tissue of ALI mice with or without MDHB gavage (left panel). Quantification of band intensity in WB (right panel). I) Analysis of cardiolipin peroxidation using the MitoCLox probe. The macrophages isolated from the lungs of ALI mice treated with or without MDHB and stained with MitoCLox. The intensity of fluorescence assessed by a microplate reader at 590 nm (non‐oxidization) and 520 nm (oxidization). The graph shows the ratio of oxidization (Ox) and non‐oxidization (Non‐Ox). J,K) Macrophage IC‐21 cells were treated with LPS followed by incubation with MDHB. Oxygen consumption rate (OCR) (J) and extracellular acidification rate (ECAR) (K) were measured using Seahorse analysis. The results showed that MDHB reduced LPS‐induced mitochondrial ECAR and increased the mitochondrial OCR in macrophages. Differences between individual groups were analyzed via two‐way ANOVA. Data are representative of three independent experiments (error bars, SD). * *p* < 0.05, ** *p* < 0.01, *** *p* < 0.001.

MDHB is a compound with antioxidant properties that has been studied for its potential effects on mitochondrial function.^[^ ^65^ ^]^ In this study, IC‐21 macrophages were cultured and stimulated with LPS for 6 h, followed by an additional 6‐h treatment with or without MDHB. The macrophages were then collected for mitochondrial function analysis. The assessment of mitochondria function in macrophages revealed that LPS treatment increased mitochondrial GTP, ATP, GSSG, and mtSOX levels in macrophages (**Figure** **6**A–D), while simultaneously decreasing GSH levels (Figure 6E). In contrast, MDHB treatment inhibited LPS‐mediated induction of GTP, ATP, GSSG, and mtSOX (Figure 6A–D), as well as LPS‐mediated reduction of GSH (Figure 6E). LPS exposure promotes mitochondrial fission which leads to an increase in the number of individual mitochondrial within a cell.^[^ ^66^ ^]^ We used TEM to observe the changes in the number of M‐Mit within ALI mouse lung tissue (Figure 6F). The results showed MDHB dramatically inhibits LPS‐induced increases in the number of M‐Mit in ALI mouse lung tissue (Figure 6G). LPS‐induced mitochondrial fission, ROS production, and impairment are triggered by modulating dynamin‐related protein 1 (DRP1) expression or phosphorylation.^[^ ^66^, ^67^, ^68^ ^]^ The WB results of macrophages in mouse lung tissue demonstrated that LPS had no effect on the expression of total DRP1 in macrophages of the ALI mouse. However, LPS significantly enhanced the phosphorylation of DRP1 (pDRP1) at Ser616, while gavage‐given MDHB significantly inhibited the LPS‐induced activation of pDRP1 (Figure 6H). DRP1 activation promotes mitochondria fission mediated by influencing the peroxidation of cardiolipin, which is an inner mitochondria membrane‐exclusive phospholipid. Next, we tested the cardiolipin oxidation using MitoCLox, which shifts from red fluorescence to green fluorescence upon oxidation. Macrophages were isolated from the lungs of ALI mice and stained with MitoCLox. The intensity analysis of fluorescence at 590 nm (non‐oxidization) and 520 nm (oxidization) suggested than MDHB blocks LPS‐induced cardiolipin peroxidation in lung macrophages (Figure 6I). Together, our data suggest that O‐Mit small molecule MDHB inhibits LPS‐mediated M‐Mit oxidative stress activation and mitochondrial dysfunction by excessive mitochondrial fission via modulating DRP1 phosphorylation and cardiolipin peroxidation.

OCR and ECAR are two key metrics used to assess cellular metabolism, particularly in the context of mitochondrial function and glycolysis. These metrics are often measured using the Seahorse XF Analyzer, which allows real‐time assessment of energy metabolism. The results generated from the Seahorse XF analyzer suggested that under LPS stimulation, mitochondrial OCR in macrophage IC‐21 cells was decreased. MDHB attenuates the impact of LPS on OCR (Figure 6J, left panel) activities such as basal respiration, ATP‐linked respiration, and maximal respiration (Figure 6J, right panel). In contrast, LPS increased mitochondrial ECAR in macrophages and MDHB alleviates the impact of LPS on ECAR (Figure 6K, left panel) activities such as glycolysis, glycolytic capacity, and glycolytic reserve (Figure 6K, right panel).

### 2.7 MDHB Modulates Mitochondrial Gene Expression by Binding Mitochondrial ND1

**Figure 7:**
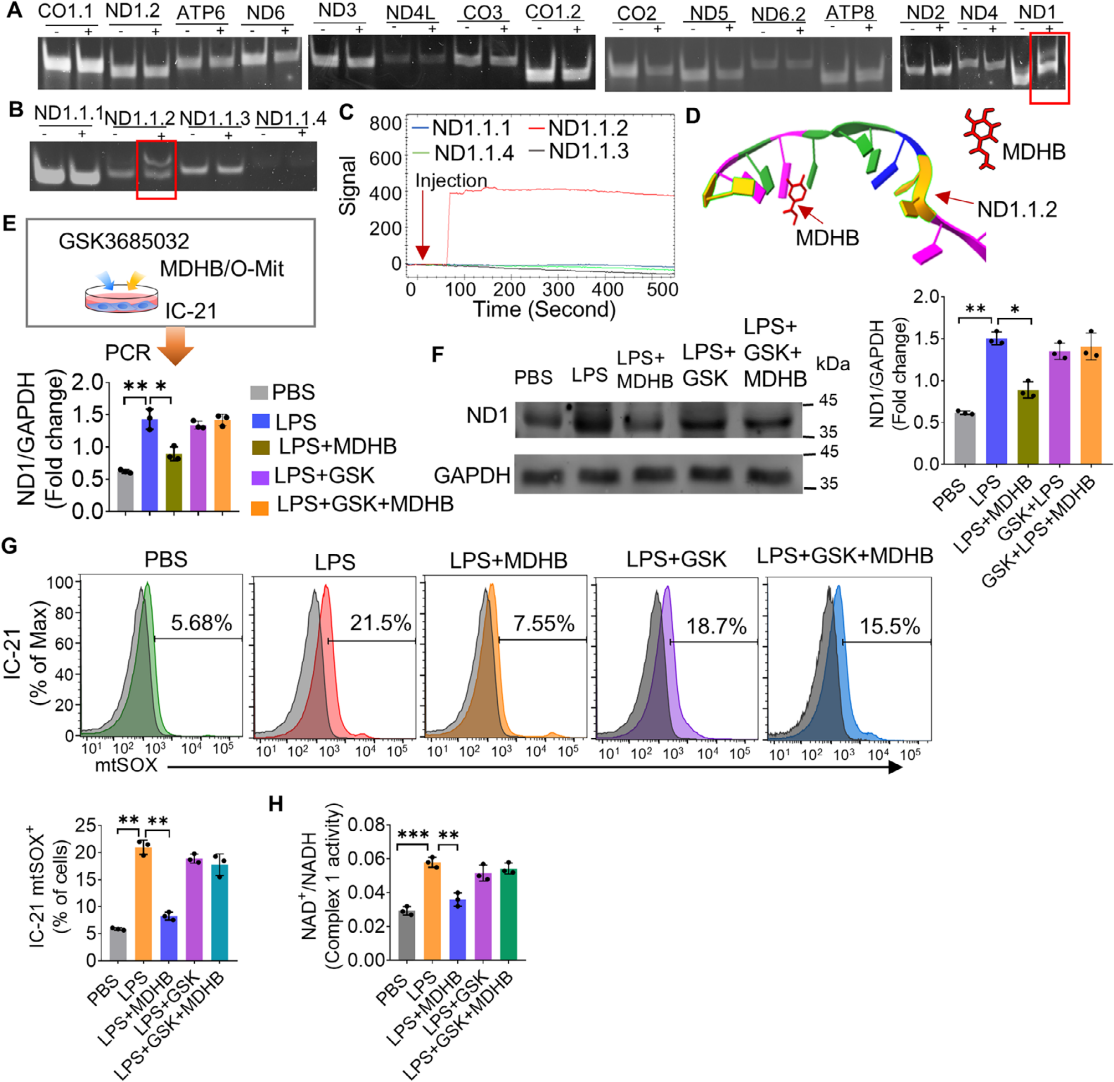
MDHB modulates mitochondrial gene expression by binding mitochondrial DNA (mtDNA). A) Mouse mtDNA was fragmented into different sizes and then incubated with or without MDHB. Agarose gel electrophoresis was used to compare the migration of fragmented mtDNA and analyze the binding of MDHB to fragmented mtDNA. The red box indicated that ND1 incubated with MDHB exhibited slower migration compared to the migration of ND1 without MDHB incubation. B) The ND1 gene of mtDNA was amplified into four fragments (50 bp per fragment). These ND1 fragments were then incubated with or without MDHB. Agarose gel electrophoresis analysis showed that ND1.1.2 incubated with MDHB exhibited slower migration (red box) compared to the migration of ND1.1.2. C) MDHB was immobilized on the Open SPR chip, and different 50 bp fragments of ND1 were injected into the chip. The signal results showed that when ND1.1.2 was injected, the signal intensity significantly increased, while no signal changes were observed when other MT‐ND1 fragments were injected. D) To verify the binding between MDHB and ND1.1.2, the 3D structures of MDHB and ND1.1.2 were submitted to the HDOCK SERVER online platform and analyzed using Chimera 1.9 software. The analysis results showed a high docking score, indicating a strong binding affinity between MDHB and ND1.1.2. E) Macrophages were treated with GSK3685032, followed by LPS stimulation for 2 h. After removing the supernatant, the cells were treated with either MDHB or GSK3685032. Macrophages were then collected for RNA extraction, and PCR was performed to analyze the expression of mitochondrial ND1. F) LPS induced an increased expression of mitochondrial ND1 protein in macrophages. However, upon binding MDHB to the mitochondrial ND1 gene, the LPS‐induced expression of mitochondrial ND1 protein in macrophages was inhibited. G) The FACS analysis results showed that MDHB significantly reduced the LPS‐induced mtSOX production in macrophages (top panel). Quantification of FACS data (bottom panel). H) Under LPS stimulation, the NAD^+^/NADH ratio in macrophages increased, indicating that the macrophages were in an oxidized state. However, MDHB treatment eliminated the LPS‐induced increase in the mtSOX and NAD^+^/NADH ratio, maintaining it at an optimal level in macrophages. Differences between percentages were analyzed with a chi‐square test. Differences between individual groups were analyzed via two‐way ANOVA. Data are representative of three independent experiments (error bars, SD). * *p* < 0.05, ** *p* < 0.01, *** *p* < 0.001.

Next, we investigated the molecular mechanism underlying how MDHB regulates mitochondrial function. We hypothesize that the small molecule MDHB in O‐Mit may bind to macrophage mtDNA with coding sequences, thereby affecting mitochondrial function. To test this hypothesis, we performed the single‐strand gel shift assay that we recently developed to test the interaction between small molecules and nuclei acids including DNA^[^ ^42^ ^]^ and RNA.^[^ ^69^ ^]^ The fragments of mouse mtDNA with coding sequences such as CO1, NADH dehydrogenases (ND), and ATPs were amplified by PCR (MmtDNA) (Table S2, Supporting Information) and incubated with or without MDHB. Whether MDHB binds to fragmented DNA was analyzed by comparison of the migration of M‐mtDNA post‐incubation using an agarose gel electrophoresis published protocol.^[^ ^70^ ^]^ The experimental results demonstrate that comparing the migration pattern of the ND1 without incubation with MDHB, the ND1 incubated with MDHB had a slower migration (**Figure** **7**A). We further PCR‐amplified four fragments (50 bp fragment^−1^) of ND1 (Table S2, Supporting Information) and then incubated these four ND1 fragments with or without MDHB. Using agarose gel electrophoresis, the results showed that comparing the migration pattern of the ND1.1.2 not incubated with MDHB, the ND1.1.2 incubated with MDHB had a slower migration (Figure 7B). To further verify the binding between MDHB and ND1 fragments using an independent approach, we used OpenSPR technology which provides label‐free interaction analysis, making it a valuable tool for researchers studying molecular interactions to measure binding kinetics and affinity without the need for labels; the molecules are preserved in their native state. We fixed MDHB onto the chip (Open SPR) and then injected different fragments of ND1 into the chip. The signal intensity was significantly increased when ND1.1.2 was injected, while no signal changes occurred when the other ND1 fragments were injected (Figure 7C). These experimental results demonstrate that the ND1.1.2 fragment specifically binds to MDHB. To investigate the interaction between MDHB and ND1.1.2, we performed computational 3D structure modeling predictions. The 3D structures of MDHB and ND1.1.2 were submitted to the HDOCK SERVER online platform and analyzed using Chimera 1.9 software.^[^ ^64^ ^]^ Based on the top three best‐fitting models, the docking score ranged from −48.03 to −42.06, and the ligand RMSD (Å) ranged from 14.63 to 58.64. We speculate that the binding of ND1.1.2 to the MDHB plays a crucial role in M‐Mit function (Figure 7D). We hypothesized that the interaction between MDHB and the mitochondrial gene ND1 in macrophages affects ND1 protein expression. To test this hypothesis, we performed qPCR and WB analysis in MDHB‐treated IC‐21 macrophages. When macrophages were treated with LPS, the expression of the mitochondrial gene ND1 and its protein was upregulated in the IC‐21 macrophages. However, MDHB treatment reduced ND1 expression at both the transcription (Figure 7E) and translation levels (Figure 7F). These experimental results suggested that MDHB inhibits the LPS‐induced upregulation of mitochondria ND1 expression in macrophages. DNA methylation is an epigenetic modification that contributes to transcriptional regulation. Recent studies confirm that mtDNA can indeed be methylated that could be dysregulated by LPS‐related inflammation especially interfering with DNA Methyltransferase 1 (DNMT1)‐mediated mtDNA methylation.^[^ ^71^ ^]^ To determine whether MDHB exerts its effects through epigenetic modifications, macrophage IC‐21 cells were treated with DNMT1 inhibitor GSK3685032^[^ ^71^ ^]^ prior to exposure with MDHB. The expression of ND1 was evaluated by WB and qPCR analysis. The results show that MDHB‐mediated downregulation of ND1 was abolished by GSK3685032 at both the transcriptional (Figure 7E) and protein levels (Figure 7F), indicating that the effects of ND1 expression are dependent on the regulation of DNA methylation.

MT‐ND1 protein is an important subunit of mitochondrial complex 1, exerting a critical role in regulating the production of mitochondrial superoxide (mtSOX) and on the NAD^+^/NADH ratio. Increased mtSOX production is linked to various diseases, including ALI. ROS acts as signaling molecules that can regulate various cellular processes, including the activation of pathways involved in inflammation. The mtSOX analysis (Figure 7G) indicated that MDHB inhibits LPS‐induced increase of mtSOX production in the macrophage IC‐21 cell, and this action of MDHB could be abolished by the DNMT1 inhibitor. The NAD^+^/NADH ratio reflects the cell's redox state, which is essential for maintaining metabolic balance and cellular function. Maintaining an optimal NAD^+^/NADH ratio is crucial for efficient metabolism and effective cellular repair mechanisms.^[^ ^72^ ^]^ We measured the NAD^+^/NADH ratio in macrophage IC‐21 cells after LPS treatment and found that the NAD^+^/NADH ratio increased in macrophages, indicating that IC‐21 cells were in an oxidized state. However, MDHB treatment eliminated the LPS‐induced increase in the NAD^+^/NADH ratio, maintaining the ratio at an optimal level in macrophages (Figure 7H). DNMT1 inhibitor GSK3685032 also repressed the effect of MDHB on the regulation of the NAD^+^/NADH ratio in macrophage (Figure 7H).

For clinical application, the potential cytotoxicity of O‐Mit was evaluated by cell proliferation and liver aminotransferase activity. The serum levels of alanine aminotransferase (ALT) and aspartate aminotransferase (AST) have been assessed as measures of drug‐related hepatotoxicity.^[^ ^73^ ^]^ The results suggested that O‐Mit has no impact on IC‐21 cell proliferation (Figure S9A, Supporting Information) and caused no significant liver damage in ALI mice when more than 20‐fold higher doses than the minimum dose that gave a therapeutic effect (Figure S9B, Supporting Information).

## 3 Discussion

In this study, we provide evidence supporting our hypothesis that plant mitochondria inhibit LPS‐induced ALI by reprogramming macrophage mitochondrial energy metabolism. Several findings from this study support this hypothesis and are summarized.

First, we demonstrated that orally administered O‐Mit traffics from the gut and reaches the mouse lung, subsequently being taken up by lung macrophages. The efficiency of uptake depends on macrophage CR1L. Additionally, we found that O‐Mit PA interacts with macrophage CR1L. This interaction is required for O‐Mit to enter macrophages. Second, once O‐Mit enters macrophages and fuses with M‐Mit, its metabolites are integrated into the M‐Mit. This integration provides an opportunity for O‐Mit methyl 3,4‐dihydroxybenzoate (MDHB) to access the macrophage mitochondrial mRNA encoding complex I subunit ND1. This action leads to the inhibition of the expression of ND1. ND1 is part of the mitochondrial complex I (NADH: ubiquinone oxidoreductase) which is the first enzyme in the mitochondrial electron transport chain. This complex plays a crucial role in cellular respiration by transferring electrons from NADH to coenzyme Q10 (CoQ10) and translocating protons across the inner mitochondrial membrane.^[^ ^74^, ^75^ ^]^ Maintaining a balanced mitochondrial NAD^+^/NADH ratio is crucial for cellular homeostasis and overall health.^[^ ^76^, ^77^ ^]^ Our results show that O‐Mit treatment can cancel the LPS‐induced imbalance of the NAD^+^/NADH ratio, decrease the levels of ROS, and decrease a variety of inflammatory cytokines. We finally demonstrated that orally administering O‐Mit can inhibit LPS‐induced mouse ALI. The LPS‐induced mouse model of ALI is widely used to study human ALI and acute respiratory distress syndrome.^[^ ^78^ ^]^ Therefore, this finding has potential clinical application for the treatment of human ALI via non‐invasive administration of O‐Mit.

MDHB has shown promising effects on mitochondrial function, particularly in reducing oxidative stress and improving mitochondrial health.^[^ ^65^ ^]^ However, the molecular mechanism underlying how MDHB affects mitochondrial function is still not clear. In this study, we demonstrated that O‐Mit MDHB is one key molecule for inhibiting the expression of ND1 by directly binding to the ND1 gene. Phenolic compounds can interact with DNA through various mechanisms, such as intercalation or binding to the minor groove, but specific studies on the binding affinity of MDHB to DNA are not well‐documented. Our findings in this study are significant because it open a new avenue for future investigations as to whether other metabolic products carried by edible plant‐mitochondria we eat daily have a role in regulating mammalian cell mitochondria activity in general. In addition, compared with the free form of plant metabolic products, metabolic products carried by plant mitochondria may differ in stability and targeting specificity. For instance, MDHB is rapidly absorbed and distributed throughout the body, including the brain, after oral administration.^[^ ^79^ ^]^ It has a short half‐life and high systemic clearance. In this study, we show that MDHB carried by O‐Mit targets lung macrophages, which is not the case for the free form of MDHB. It is likely that MDHB carried by O‐Mit has a different tissue distribution from the free form of MDHB in vivo.

Modulation of the expression of complex I genes benefits health and the lifespan in mice and humans. ND1 is one of the subunits of complex I, and the downregulation of ND1 expression can attenuate complex I activity.^[^ ^80^ ^]^ The overactivity of mitochondrial Complex I can contribute to the development and progression of various lung diseases,^[^ ^81^ ^]^ including ALI and chronic obstructive pulmonary disease. Overactive Complex I can lead to excessive production of ROS, which can cause oxidative damage to lung tissues. This oxidative stress is a significant factor in the pathogenesis of inflammatory lung diseases. Increased ROS production from overactive Complex I can trigger inflammatory pathways, leading to the recruitment of immune cells and the release of pro‐inflammatory cytokines. This inflammation can exacerbate lung injury and contribute to diseases like ALI. Targeting Complex I activity and reducing oxidative stress may offer therapeutic benefits for treating lung diseases. For example, metformin, a complex I inhibitor, is used to treat aging disorders,^[^ ^82^ ^]^ diabetes,^[^ ^83^ ^]^ and cancer.^[^ ^84^ ^]^ Inhibition of mitochondrial complex I has shown potential in delaying brain aging and mitigating neurodegenerative diseases. Partial inhibition of complex I can improve energy homeostasis, reduce oxidative stress and inflammation, and enhance cognitive function in models of Alzheimer's disease.^[^ ^85^ ^]^ Collectively, our data presented in this study suggest that O‐Mit MDHB‐mediated targeting mitochondrial ND1 could be a promising strategy for promoting lung health and inhibition of the onset of inflammation‐related lung conditions thereby opening a new avenue for plant mitochondria transfer therapy.

Moreover, mitochondria are at the center of metabolism plasticity and have evolved to become multifaceted signaling organelles.^[^ ^86^ ^]^ Our findings show that edible plant mitochondria can alter M‐Mit complex I activity. This result provides a foundation for further investigating whether other factors besides the identified MDHB carried by O‐Mit can shift its recipient cell metabolic flux to match extrinsic or intrinsic challenges and modify cellular functions to match cell‐type‐specific needs by modulating mitochondria complex I activity. Therefore, the findings we report in this study highlight the intricate multifaceted, and interlinked modes of plant‐mammalian cell mitochondrial inter‐species communication within cells.

Our results presented in this study show that MDHB treatment led to decreasing mitochondria ROS and SOX expression, and inhibition of LPS‐mediated induction of inflammatory cytokines. Several downstream pathways regulated by ROS can lead to the induction of inflammatory cytokines. For example, excess ROS can activate the NLRP3 inflammasome, a multi‐protein complex that plays a crucial role in the innate immune response.^[^ ^87^ ^]^ Activation of the NLRP3 inflammasome leads to the maturation and release of pro‐inflammatory cytokines such as IL‐1β. ROS can also activate the NF‐κB (nuclear factor kappa‐light‐chain‐enhancer of activated B cells) signaling pathway.^[^ ^88^ ^]^ NF‐κB is a transcription factor that regulates the expression of various pro‐inflammatory genes. The mitogen‐activated protein kinase (MAPK) signaling pathway can be activated by ROS.^[^ ^89^ ^]^ The MAPK pathway includes several kinases such as ERK, JNK, and p38, which regulate the production of inflammatory mediators and cytokines. These pathways collectively contribute to the inflammatory response, leading to the production and release of various pro‐inflammatory cytokines and mediators.^[^ ^40^ ^]^ The resulting inflammation can further exacerbate mitochondrial dysfunction, creating a vicious cycle of oxidative stress and inflammation. These pathways have dual roles and highlight the importance in maintaining cellular homeostasis and the use of plant mitochondria as potential therapeutic agents for various diseases. Directed delivery of MDHB to dysregulated mitochondria of inflammatory cells and no other types of cells is critical. The data presented in this study are significant since MDHB carried by O‐Mit is predominately delivered to the mitochondria of lung macrophages and downregulates the production of lung macrophage ROS.

In summary, in this study, we demonstrated that several key challenges in mammalian mitochondrial transfer therapy can be addressed by using edible P‐Mit.

1) The exact mechanisms by which transplanted mammalian mitochondria exert therapeutic effects are still not fully understood. 2) Mammalian mitochondria are sensitive to pH and oxidative stress which can impair their therapeutic potential during isolation, storage, and delivery.^[^ ^90^, ^91^ ^]^ 3) Efficient and targeted delivery to specific tissues or cells remains a challenge. 4) Transplanted mitochondria may trigger immune responses, especially if they are allogeneic (from another individual);^[^ ^92^ ^]^ and 5) Widespread clinical use will require cost‐effective and scalable solutions, and the procedures for mitochondrial isolation, purification, and transplantation are technically demanding and expensive. There is no consensus on the best tissue sources, isolation protocols, or quality control standards for therapeutic mitochondria. Obtaining high‐quality donor mitochondria is a major hurdle, and current methods (e.g., direct injection, cell‐mediated transfer, or vesicle‐based delivery) vary in efficacy, safety, and scalability.

Unlike mammalian mitochondria, several unique features of plan mitochondria can be utilized to overcome the challenge of mammalian mitochondria‐based transfer therapy with the following features. 1) Plant mitochondria have evolved distinct biochemical and structural adaptations to cope with environmental stress.^[^ ^93^, ^94^, ^95^ ^]^ While plant mitochondria are not naturally exposed to gut environments, their robust stress‐adaptive systems—including alternative respiration, signaling flexibility, and metabolic plasticity ‐ equip them to survive in hostile or fluctuating environments that share features with gut‐like conditions (e.g., low oxygen, high ROS, acidic pH). A study published^[^ ^96^ ^]^ using cardiolipin, a mitochondria marker, found that plant cardiolipin contains more unsaturated fatty acid chains compared to mammalian cardiolipin. This affects how cardiolipin interacts with mitochondrial proteins, particularly in respiratory Complex I, and contributes to membrane flexibility and protein stability of mitochondria. In *Arabidopsis thaliana*, cardiolipin plays a dominant role in mitochondrial fission, which is different from its role in mammals where it more strongly influences fusion.^[^ ^97^ ^]^ 2) Plant mitochondria are stable in variable pH conditions.^[^ ^98^, ^99^ ^]^ Plant mitochondria are more tolerant of acidic environments due to their adaptation to variable pH conditions in soil, and this adaptation is applicable to variable pH in the gut. In this study, we provided additional evidence to show that O‐Mit is stable in the gut and lungs. The FACS analysis shows that JC‐1 labeled O‐Mit were not shifted from the red PE channel (polarized) to the green FITC channel (depolarized) in gastric fluid and gut fluid. The oxygen flux assay shows no significant impact of oxygen consumption on O‐Mit exposed to gut fluid. These data indicated that O‐Mit was functional and intact under gastric fluid and gut conditions. PCR analysis also suggested little influence of gastric and gut fluid on O‐Mit mtDNA integrity in the gut environment. In contrast, mammalian mitochondria isolated from gut epithelial cells are not stable. 3) There is immune tolerance to edible plant mitochondria. Diets including edible plants exert immune tolerance resulting from continuous surveillance of intestinal contents.^[^ ^20^ ^]^ Mitochondria are naturally occurring organelles in plant cells. Because they are derived from edible plants that humans have consumed for millennia, the human body is already accustomed to their molecular components. Therefore, this natural compatibility contributes to their low immunogenicity. In addition, edible plant mitochondria contain bioactive compounds like microRNAs, lipids, and metabolic products like MDHB that have anti‐inflammatory or immunomodulatory effects, further reducing the likelihood of an immune reaction. Plant‐derived foods elicit immune tolerance by triggering the action of regulatory T cells (Tregs) in the gut.^[^ ^20^, ^21^ ^]^ In this study, we also provide evidence that O‐Mit can induce IL‐10 and TGF‐β in intestinal FoxP3^+^CD4^+^ T cells via TGF‐β released from O‐Mit‐treated macrophages. Our data is also supported by others indicating that TGF‐β not only serves as a hallmark suppressive cytokine of FoxP3⁺CD4⁺ Tregs but also participate in reinforcing their own expression through paracrine signaling loops.^[^ ^100^ ^]^ While IL‐10 is a key effector cytokine of Tregs and can be upregulated in response to TGF‐β signaling.^[^ ^101^ ^]^ Our findings set up a foundation for future studies focusing on isolating and characterizing the O‐Mit bioactive components responsible for Treg induction; 4) Unlike Mammalian mitochondria, plant mitochondria can be produced on a large scale with controlled quality at a very low cost. The quality of the isolated plant mitochondria used for therapy can be evaluated using JC‐1 and NAD^+^/NADH‐based function assays. Mitochondria can be stored at −80 °C, and their JC‐1 and NAD+/NADH‐based activity can be tested prior to the use of batches. Only P‐Mit, such as O‐Mit, with activity greater than 90% compared with that of freshly isolated mitochondria should be used. 5) Inflammation plays a central role in many different types of diseases including neuronal diseases, cardiovascular diseases; infectious diseases (such as sepsis, COVID‐19), autoimmune diseases; cancer; and metabolic disorders. In this study, we show that O‐Mit can inhibit lung inflammation. Mitochondria from a variety of plants contain a diversity of contents such as lipids, proteins, small RNAs, etc., which could determine the targeted tissue and anti‐inflammatory effect through a variety of molecular mechanisms. Given O‐Mit is also distributed to the liver, the potential cytotoxicity of O‐Mit was evaluated. The serum levels of alanine aminotransferase (ALT) and aspartate aminotransferase (AST) have been used to assess drug‐related hepatotoxicity.^[^ ^73^ ^]^ In our study, the results suggested that O‐Mit caused no significant liver damage in ALI mice at even higher doses than a therapeutic dose.

Therefore, it is conceivable that besides using P‐Mit in the ALI mouse model we tested in this study, edible plant mitochondria could be used for the treatment of other inflammation‐related types of diseases. This would most likely depend on the plant mitochondria selected based on their tissue distribution in the recipient.

The tissue distribution of mitochondria from a variety of plants could be determined by several factors.

*Size of the P‐Mit*: Particle size plays a crucial role in determining the biodistribution of nanoparticles and larger particles in the body. Nanoparticles between 100 and 200 nm are efficiently cleared by Kupffer cells in the liver, while larger particles (>1 µm) are more likely to be trapped in the lungs.^[^ ^102^, ^103^ ^]^ Given mitochondria are heterogeneous in size ranging between 500 nm–2 µm,^[^ ^104^ ^]^ we found that there is a subset of O‐Mit that preferentially accumulates in the lung.

*Lipid composition of P‐Mit*: Macrophage uptake phospholipid‐composed liposome in a lipid dependent manner. In this study, we identified that macrophages in the lungs uptake of O‐Mit is attributed to the interaction of macrophage membrane protein CR1L with phosphatidic acid (PA) of the O‐Mit. This high abundance of PA partially explains why O‐Mit distributes to the lungs more than garlic‐Mit and soybean‐Mit.

*Inflammation*: Inflammation increases endothelial cell activation and immune cell recruitment in the lungs^[^ ^105^ ^]^ leading to an increase in vascular permeability. Lung inflammation promotes the release of inflammatory factors from immune cells. The inflammatory factors include cytokines that can cause endothelial cell disruption and increase the permeability of the lung vasculature.^[^ ^106^ ^]^ To test if LPS intranasal injection‐induced lung inflammation enhances P‐Mit retention in the lungs, we estimated the distribution of P‐Mit/DiR in naïve mice without LPS treatment. Our image analysis showed that P‐Mit/DiR in the lungs is significantly lower in naïve mice compared to ALI mice. Collectively, our experimental evidence suggested that the distribution of P‐Mit in lungs is influenced by multiple factors including size, mitochondria composition, and inflammatory conditions.

Our findings presented in this study also further open an avenue for studying the molecular mechanisms underlying how O‐Mit mitochondria penetrate the intestinal mucus layer and traverse epithelial tight junctions. There are several plausible mechanisms supported by existing literature: 1) the gut mucus layer is composed of negatively charged mucins,^[^ ^107^ ^]^ which form a selective barrier. Positively charged particles tend to adhere to mucins via electrostatic attraction, limiting their mobility. In contrast, negatively charged particles like O‐Mit may experience electrostatic repulsion, facilitating their diffusion through the mucus layer. This charge‐based selectivity has been demonstrated in nanoparticle delivery systems^[^ ^108^ ^]^ and may similarly apply to O‐Mit; 2) Tight junctions are typically impermeable but can be transiently modulated by bioactive peptides or surface ligands.^[^ ^109^ ^]^ O‐Mit may carry such ligands capable of influencing the expression or conformation of tight junction proteins (e.g., claudins, occludin, ZO‐1), thereby enabling paracellular passage; 3) Additionally, inflammatory signals present in disease states can loosen junctional integrity, further facilitating translocation of O‐Mit; and 4) Once past the epithelium, O‐Mit may enter the portal circulation. While the liver typically acts as a clearance organ via Kupffer cells, certain particle characteristics—such as shape, surface composition, and lipid content—can influence hepatic uptake.^[^ ^100^ ^]^ Edible plant mitochondria may possess features that allow partial evasion of liver clearance, enabling delivery to distal organs such as the lungs.

## 4 Experimental Section

### Mice

Six to eight‐week (w)‐old male specific‐pathogen‐free (SPF) C57BL/6 mice were purchased from the Jackson Laboratory (Bar Harbor, ME). All mice were housed under SPF conditions. Animal care was performed following the Institute for Laboratory Animal Research (ILAR) guidelines, and all animal experiments were conducted in accordance with protocol (#24 374) approved by the University of Louisville Institutional Animal Care and Use Committee (Louisville, KY). The mice were acclimated for at least 1 week before any experiments were conducted.

### Cell Culture

The C57BL/6 murine macrophage IC‐21 cell line was purchased from the American Type Culture Collection (ATCC, TIB‐186) and grown in tissue culture plates with RPMI 1640 medium (Life Technologies) supplemented with 10% heat‐inactivated fetal bovine serum (Gibco, FBS), 100 U mL^−1^ penicillin, and 100 µg mL^−1^ streptomycin at 37 °C in a 5% CO_2_ atmosphere.

### Isolation of Leukocytes from Mouse Tissues

To isolate the leukocyte, transcardiac perfusion was conducted via the inferior vena cava with perfusion buffer (Ca^2+^‐Mg^2+^free HBSS containing 0.5 mm EGTA, 10 mm HEPES, and 4.2 mm NaHCO_3_; pH 7.2). The mouse tissues such as lungs and small intestine were removed, homogenized, and dissociated gently using a tissue dissociator (Miltenyi Biotec gentleMACS dissociator). Briefly, the mouse tissues were incubated with 1000 µm of collagenase 1 at 37 °C for 15 min followed by transferring the mixture to the C Tube with collagenase 1. The lung tissue was dissociated at room temperature for 10 min using a tissue dissociator. The resuspended cell suspension was passed through a MACS Smart Strainer and washed with 10 mL of HBSS. Cells were resuspended in 70% Percoll^[^ ^41^ ^]^ (Sigma‐Aldrich), overlayed with 37% and 30% Percoll, and centrifuged at 500 × g for 20 min at 22 °C. Enriched leukocyte populations were recovered at the 70–37% Percoll interface. The leukocyte cell layer was carefully collected with a pipette and washed three times with PBS.

### Isolation of Mitochondria from Plants and Macrophage Cells

Onion, soybeans, and garlic were purchased from a supermarket, peeled after disinfecting with 70% ethyl alcohol (Sigma‐Aldrich, ACS reagent), and the plant mitochondria were isolated employing minor modifications of a previously described method.^[^ ^23^, ^24^ ^]^ The plants were homogenized in a low‐speed blender with homogenization buffer A (0.4 m sucrose, 1 mm EDTA, 25 mM MOPS KOH (pH 7.8), 10 mm tricine, 8 mm cysteine, 0.1% BSA, and 1% PVP‐40) for 1–2 min. The juice was collected after net filtration and diluted with homogenization buffer. The filtered homogenate was then centrifuged for 10 min at 1000 × g. The supernatants were collected, and the organelles were sedimented by centrifugation at 12 000 × g for 30 min at 4 °C. The pellets were washed with wash buffer (0.4 m sucrose, 1 mm EDTA, 10 mm MOPS–KOH, pH7.2, and 0.1% BSA). The washed organelles were further purified by centrifugation at 12 000 × g for 30 min at 4 °C in a sucrose density gradient (0.6 to 1.8 m sucrose in 10 mm Tri‐Cl, pH 7.2, 1 mm EDTA, 0.1% BSA). After centrifugation, the mitochondria were collected from the 1.25 to 1.35 m sucrose band and washed with PBS. The isolated plant‐mitochondria were suspended in a maintenance buffer (0.4 m sucrose, 1 mm EDTA, 10 mm MOPS–KOH, pH7.2, and 0.1% BSA)^[^ ^23^ ^]^ modified by adding 10 mm disodium hydrogen phosphate (Na_2_HPO4), 1 mm sodium pyruvate, EGTA replace EDTA, 0.01 mg mL^−1^ mitochondrial stabilizer Egb761^61^ to preserve mitochondrial integrity and activity.

To extract mitochondria from lung macrophages in mice or macrophage IC‐21 cells, F4/80 beads were used to pull‐down macrophages from the lung leukocyte. The mouse macrophages or macrophage IC‐21 cells were homogenized using a Teflon‐glass homogenizer in buffer B (Sucrose 0.32 m, EDTA 1 mm, Tris‐HCl 10 mm, pH 7.4).^[^ ^110^ ^]^ The homogenate was centrifuged for 10 min at 1000 × g, and the supernatants were collected. The crude organelles were obtained by centrifugation at 12 000 × g for 30 min at 4 °C. The pellets were washed with buffer B followed by sucrose density gradient centrifugation.^[^ ^110^ ^]^ The mitochondrial band was collected, washed, and resuspended in modified maintenance buffer using the same method described above. All containers were autoclaved and reagents filtered using a 0.22 µm filter. Purified mitochondria were visualized under a Zeiss EM 900 electron microscope using a previously described method.^[^ ^39^ ^]^

### Labeling of Mitochondria and Liposomes with Fluorescent Dye

Plant mitochondria and liposomes were labeled with lipophilic tracers including DiR (Em, 780 nm), DiO (Em, 501 nm), and DiI (Em, 565 nm) (Invitrogen) in accordance with the manufacturer's instructions. The stock solution of the tracers was prepared in dimethylsulfoxide (DMSO) at 1 mg mL^−1^. After a wash with PBS, mitochondria (10 mg) and liposomes (10 mg) were suspended in 250–500 µL of PBS with 2–4 µl of stock solution and subsequently incubated for 30 min at room temperature. After centrifugation for 30 min at 12 000 × g, labeled mitochondria and liposomes were resuspended and washed three times with PBS.

### Plant Mitochondria Distribution In Vivo

Purified soybean mitochondria (S‐Mit), onion mitochondria (O‐Mit), and garlic mitochondria (G‐Mit) were labeled with DiR dye. The DiR‐labeled S‐Mit, O‐Mit, and G‐Mit were administered to LPS‐induced ALI C57BL/6 (ALI) mice (*n* = 5) by oral gavage at a dose of 100 µL (10 mg mL^−1^). After 6 h, transcardiac perfusion was conducted via the inferior vena cava using perfusion buffer (Ca2+‐Mg2+free HBSS containing 0.5 mm EGTA, 10 mm HEPES, and 4.2 mm NaHCO3; pH 7.2). Organs and tissues were collected, and the DiR‐labeled plant mitochondria in the mice organs and tissues were visualized using an Odyssey CLx Imaging System (Licor Biosciences).

### Zeta Potential and Refractive Index

To measure the Zeta potential of the S‐Mit, O‐Mit, and G‐Mit, the Zeta View particle tracking analyzer instrument was used. The S‐Mit, O‐Mit, and G‐Mit were suspended in deionized water, and the concentration was adjusted to 1 × 10^8^ mitochondria mL^−1^. To measure the Zeta potential of the S‐Mit, O‐Mit, and G‐Mit suspensions were injected into the Particle mtrix Zeta View analyzer using a 1 mL syringe. The S‐Mit, O‐Mit, and G‐Mit were diluted to different concentrations (6.2, 12.5, 25, 50, and 100 mg mL^−1^) using PBS, and the refractive index of mitochondria was measured with a refractometer (Leica MARK II PLUS Abbe Refractometer).

### Measurement of Rectal Temperature in Mice

C57BL/6 mice (*n* = 5) were intranasally administered 50 µg mouse^−1^ of LPS. After six hours, the mice were gavage‐given 1 mg mouse^−1^ of O‐Mit or 0.5 mg mouse^−1^ of MDHB. Rectal temperature was measured at 0, 2, 4, 6, 8, 10, and 12 h. At each designated time point, the mice were gently restrained, and a specialized temperature probe with a diameter of ≈1.5 mm was lubricated and slowly inserted ≈2 cm into the rectum. The probe was held in place until the temperature reading stabilized and the value was recorded. After each measurement, the probe was wiped and disinfected with 75% ethanol.

### The Dry/Wet Weight Ratio of Mouse Lung Tissue

To assess pulmonary edema in mice, the lung wet/dry weight ratio in different treatment groups was measured. After different treatments, the mice were euthanized, and the lung tissue was collected. The lung tissue was rinsed three times with PBS, and the surface liquid was absorbed using sterile filter paper before weighing the wet weight of the lung tissue. Then, the lung tissue was placed in a 60 °C oven for 24 h until a constant weight was reached, after which the dry weight was measured. The lung Wet/Dry (W/D) weight ratio was recorded using the following formula: W/D ratio = Wet weight/Dry weight.

### Preparation of Transmission Electron Microscopy (TEM) Samples

C57BL/6 murine lung tissue and mitochondria were fixed for 2 h at 22 °C in 2% paraformaldehyde (Electron Microscopy Science, PA) in PBS, followed by 1% glutaraldehyde (Electron Microscopy Science, PA) for 30 min at 22 °C. Fifteen microliters of fixed samples or brain tissue sections (100 nm) were put on 2% agarose with formvar/carbon‐coated copper grids on top and allowed to absorb for 5–10 min. The grids with adherent mitochondria were fixed in 2% paraformaldehyde in PBS for 10 min followed by extensive washing in PBS. Negative contrast staining was performed with 1.9% methyl cellulose and 0.3% uranyl acetate for 10 min. The grids with negatively stained mitochondria were dried before observation under a Zeiss EM 900 electron microscope.

To label O‐Mit with the gold‐Triangle nanoparticles (GNTs) for TEM analysis, isolated O‐Mit (1 mg mL^−1^) in 1× HBSS was incubated with 10 µg of N‐hydroxysuccinimide (NHS)‐terminated gold‐triangle nanotriangles (Nanopartz, C1T‐625‐TNHS‐DRY‐0.5) in pH 8 at 30 °C for 12 h. After centrifuged at 10 000 × g for 15 min, the gold‐Triangle nanoparticle‐labeled O‐Mit were washed three times with HBSS (pH = 7) and resuspended it in 2.5% glutaraldehyde buffer in 0.1 m sodium cacodylate buffer pH 7.4 (Electron Microscopy Sciences, 16527‐20) overnight at 4 °C for TEM analysis or stored in −20 °C for later experimental use.

To label mouse mitochondria lung tissue in situ with the gold‐sphere nanoparticles (GNPs) for TEM analysis, the lung tissue section was loaded on 2% agarose‐formvar/carbon‐coated copper grids for 30 min. The grids were washed three times with 0.02 m glycine in PBS and then treated with a blocking solution of 1% bovine serum albumin (BSA) in phosphate‐buffered saline (PBS). The grids were then floated for 1 h on droplets of anti‐Tom20 antibody (1:200) in blocking solution. After a wash with 0.1% BSA in PBS, the grids were incubated for 30 min with protein G or protein A‐conjugated 10‐nm‐diameter Gold‐Sphere particles (Cytodiagnostics, Canada) diluted 1:40 in blocking solution. After one wash in PBS, the grids were fixed with 1% glutaraldehyde, washed with distilled water, and then stained with 1% ammonium molybdate. After excess ammonium molybdate was removed from the grid by washing three times using PBS, images were visualized using a Thermo‐Fisher TEM Tecnai Spirit at 80 kV, and images were collected with an AMT XR60 digital camera.

### Extraction of Mitochondrial Lipids

To extract the lipids from isolated mitochondria, 1‐part volume (1.6 mL) mitochondria was mixed with 1.25‐part chloroform (2 mL) and 2.5‐part methanol (4 mL) in a glass tube and shaken well. An additional 1.25‐part (2 mL) chloroform and 1.25‐part (2 mL) H_2_O were added and mixed well. The mixture was centrifuged at 2000 rpm for 10 min at room temperature, and the lower phase transferred to a clean glass tube.^[^ ^111^ ^]^ All of the collections and the lipids were dried by nitrogen (2 psi) and stored at −80 °C.

### Isolation of Gastric and Gut Fluids from Mice

C57BL/6J mice (6–8 weeks) were fast for 12 h. After euthanasia, the abdominal cavity was opened to expose the gastrointestinal tract. The esophagus and pylorus were ligated using sterile surgical thread, and the entire stomach was carefully removed using sterile scissors. The stomach tissue was placed in sterile PBS to rinse the outer surface. The stomach was then opened along the body region, and the gastric contents were flushed out using 100 µL of PBS. The collected gastric contents were centrifuged at 1000 × g for 15 min at 4 °C. The supernatant was collected for use or stored at −80 °C for future experiments. A segment of the mouse small intestine was ligated using sterile surgical thread and excised using sterile scissors. The exterior of the intestinal segment was rinsed with sterile PBS. Using a sterile syringe, 1 mL of sterile PBS was injected into one end of the intestinal segment to flush the lumen. The intestinal lavage fluid (gut fluid) was collected and centrifuged at 1000 × g for 15 min at 4 °C. The supernatant was collected for use or stored at −80 °C for future experiments.

### Lipidomic Analysis with Liquid Chromatography‐Mass Spectrometry (LC‐MS)

Lipid samples extracted from mitochondria were submitted to the Lipidomics Research Center, Kansas State University (Manhattan, KS) for analysis using a method previously described.^[^ ^112^ ^]^ In brief, the lipid composition was determined using triple quadrupole MS (Applied Biosystems Q‐TRAP, Applied Biosystems, Foster City, CA). The data were reported as the percentage of each lipid within the total signal for the molecular species determined after normalization of the signals to internal standards of the same lipid class.

### Extraction of Mitochondrial Metabolites

To extract metabolites from purified O‐Mit, the purified O‐Mit (2 mg mL^−1^) was centrifuged at 12 000 × g for 30 min at 4 °C, and the pellet was collected. One milliliter of cetyltrimethylammonium bromide (CTAB) lysis buffer was added to the mitochondrial pellet, followed by vigorous vortexing for 2 min. After centrifugation at 12 000 × g for another 30 min, the supernatant was collected and stored at −80 °C.

### Coomassie Blue Staining

The O‐Mit proteins were separated on a 10% sodium dodecyl sulfate–polyacrylamide gel electrophoresis (SDS‐PAGE) gel. The SDS‐PAGE gel was fixed and stained for imaging using 0.1% Coomassie Brilliant Blue R‐250 (BioRad).

### Preparation of Liposome

One milligram of lipid was dissolved in 2 mL of anhydrous ethanol. The lipid solution was filtered using a 0.45 µm syringe PES filter (Santa Cruz Biotechnology, sc‐516779). Deionized water was filtered using a 0.45 µm syringe PES filter (Santa Cruz Biotechnology, sc‐516779) to obtain the sterile water solution. The liposomes were generated using a NanoGenerator (PreciGenome LLC) according to the manufacturers’ instructions. The lipid solution and filtered deionized water were supplied to well A and well B with a flow rate of 1 and 0.5 mL min^−1^, respectively according to the operating instructions. The prepared liposomes were collected from well C.

### Mouse Cytokines Protein Array

To investigate the effects of O‐Mit on the regulation of cytokine expression in the lung tissue, ALI mice were gavaged with 100 µL O‐Mit (10 mg mL^−1^). The mice were euthanized for lung tissue collection. Lung tissue extracts were prepared using modified radioimmunoprecipitation assay (RIPA) buffer (Sigma) with the addition of protease and phosphatase inhibitors (Roche). Cytokine protein analysis was performed using the Proteome Profiler Mouse XL Cytokine Array Kit (R&D Systems, ARY028). Quantification of the spot intensity in the arrays was conducted with background subtraction using ImageJ.

### Mouse Lung Inflammatory PCR Array

Total RNA was extracted from the isolated macrophages using the RNeasy Mini Kit (Qiagen, Germany), and the concentration and purity of the extracted RNA were measured using a Nano Drop spectrophotometer (Thermo Fisher Scientific). One microgram of total RNA was treated at 42 °C for 5 min, followed by reverse transcription at 37 °C for 60 min. The reaction was then terminated by heating at 95 °C for 5 min. Inflammatory gene expression analysis was performed using the Qiagen RT^2^ Profiler PCR Array (Mouse Inflammatory Response and Autoimmunity, Cat. No. PAMM‐077Z). The resulting cDNA was mixed with RT^2^ SYBR Green ROX qPCR Mastermix (Qiagen, Cat. No. 330 522) and RNase‐free water to a final volume of 25 µL per well. The reaction mixture was loaded into the PCR array plate. Real‐time quantitative PCR was carried out using the ABI StepOnePlus Real‐Time PCR System. CT values for each well were obtained using the qPCR instrument, and data analysis was performed using the Qiagen online data analysis platform.

### Mitochondrial Guanosine Triphosphate (GTP) Analysis

GTP was assessed using the GTPase‐Glo assay (Promega) according to the manufacturers’ instructions. Cells (1 × 10^4^) were plated in a 96‐well plate and incubated overnight in a cell culture incubator. The cells were subjected to different treatments and further incubated for 6 h. After removing the supernatant, 50 µL of cell culture medium and 50 µL of 2X GTP solution were added, followed by incubation at room temperature for 80 min. Subsequently, 50 µL of GTPase‐Glo reagent was added, gently mixed, and incubated with shaking at room temperature for 30 min. Finally, 20 µL of detection reagent was added, and the plate was incubated at room temperature for 6 min. Without washing, luminescence intensity was measured using a BioTek microplate reader (Agilent).

### Adenosine Triphosphate (ATP) Analysis

The ATP was assessed using an ATP luminescence assay kit (Dojindo) according to the manufacturers’ instructions. In a 96‐well plate, 1 × 10^4^ cells were plated and incubated overnight in a cell culture incubator. The cells were subjected to different treatments and further incubated for 6 h. ATP standard solutions were added to individual wells of a 96‐well microplate, followed by the addition of 100 µL of working solution per well. The plate was then incubated at 25 °C for 10 min. Without washing, luminescence intensity was measured using a BioTek microplate reader (Agilent).

### Glutathione (GSH) and Glutathione Disulfide (GSSG) Analysis

The GSH and GSSG were measured using detection assay kits (Dojindo) according to the manufacturers’ instructions. Macrophage IC‐21 cells (1 × 10^7^) were collected by centrifugation at 300 × g for 5 min at 4 °C. The pelleted cells were collected and washed with PBS. Eighty microliters of 10 mmol L^−1^ HCl was added to the cells and the cells were lysed by two cycles of freezing and thawing. Twenty microliters of 5% SSA was added to the lysed cells followed by centrifugation at 8000 × g for 10 min. The supernatant was transferred to a clean tube, and ddH_2_O added to reduce the SSA concentration to 0.5% for the assay. The absorbance was read at 405 or 415 nm using a BioTek microplate reader (Agilent).

### Mitochondrial Reactive Oxygen Species (ROS) Analysis

The ROS was assessed using MitoBright ROS Deep Red (Dojindo) according to the manufacturers’ instructions. In a 96‐well black plate, 2 × 10^5^ cells were plated and incubated with 100 µL 1× Hanks' Balanced Salt Solution (HBSS) containing 10 µmol L^−1^ MitoBright ROS Deep Red at 37 °C for 30 min. After washing with 1× HBSS, 100 µL HBSS containing Antimycin (stimulator, 10 µmol L^−1^) was added into wells of the plate for 15 min at 37 °C. Without washing, the fluorescence intensity (Ex: 535–565 nm and Em 660–690 nm) was measured using a BioTek microplate reader (Agilent).

### Mitochondrial Superoxide (mtSOX) Analysis

The mtSOX was estimated using MitoSOX Mitochondrial Superoxide indicator (M36008, Invitrogen) according to the manufacturer's instructions. In a 96‐well black plate, 2 × 10^5^ cells were plated and incubated with 100 µL 1× Hanks' Balanced Salt Solution (HBSS) containing 500 nmol L^−1^ MitoSOX Mitochondrial Superoxide indicator at 37 °C for 30 min. After washing with 1× HBSS, 100 µL HBSS containing Antimycin (10 µmol L^−1^) was added into the plate at 37 °C for 15 min. Without washing, the fluorescence intensity (Ex: 396 nm and Em 610 nm) was measured using a BioTek microplate reader (Agilent).

### Analysis of Oxidized Cardiolipin Using MitoCLox

Macrophage cells were plated into 96‐well plates (1 × 10^5^ well^−1^) and incubated with 200 nm MitoCLox (Lumiprobe) at 37 °C for 30 min. After washing with PBS, the intensity of fluorescence absorbance was measured with a BioTek microplate reader (Agilent) at 590 nm (non‐oxidization) and 520 nm (oxidization). The oxidized cardiolipin was indicated using ratiometric measurement (520/590 nm).

### Thin‐Layer Chromatography (TLC) Analysis

TLC analysis was performed using a previously described method.^[^ ^113^ ^]^ Briefly, high‐performance thin‐layer chromatography (HP‐TLC) plates (silica gel 60 with a concentrating zone, 20 cm × 10 cm; Merck) were used for separation. After the lipid and metabolite samples extracted from onion mitochondria were concentrated, the samples were spotted onto the chromatography plate and developed using a chloroform/methanol/acetic acid (190:9:1, by volume) solvent system. After development, the plate was air‐dried and placed in a glass container with iodine vapor at room temperature for 10 min. The lipid and metabolite bands were imaged using an Odyssey Scanner (Licor Bioscience, Lincoln NE).

### Mitochondrial NAD^+^/NADH Ratio Assay

Mitochondrial NAD^+^/NADH ratios were assessed using a NAD^+^/NADH Assay Kit (Sigma‐Aldrich, MAK460) according to the manufacturers’ instructions. Macrophages (2 × 10^5^) were suspended in 100 µL of NAD^+^ or NADH Extraction Buffer and incubated at 60 °C for 5 min. The extraction reaction was neutralized with Opposite Buffer and subsequently centrifuged at 14 000 × g for 10 min at 4 °C. Fifty microliters of supernatant was mixed with the same volume of fresh Working Reagent in a 96‐well black plate and the fluorescence intensity measured (Ex: 530 nm and Em 585 nm) using a BioTek microplate reader (Agilent).

### Analysis of Cell Energic Metabolism Using Seahorse Assay

To assess mitochondrial energy metabolism, both oxygen consumption rate (OCR) and extracellular acidification rate (ECAR) were carried out to monitor mitochondrial respiration and glycolysis in cells using selective substrates/inhibitors at different metabolic states. The OCR and ECAR measurements were performed with the Seahorse XF Cell Mito Stress Test Kit (Agilent) and Seahorse XF Glycolysis Stress Test Kit (Agilent) using the XFe96 Analyzer (Agilent), according to the manufacturer's instructions. Briefly, macrophage IC‐21 cells were seeded into Seahorse XF Pro microplates at a density of 1.5 × 10^4^ cells per well and cultured at 37 °C overnight. The next day the medium was replaced with low‐buffered XF assay minimal DMEM (Agilent) to equilibrate for 1 h at 37 °C in a non‐CO_2_ incubator. Seahorse XF analysis was performed at 37 °C simultaneously measuring Oxygen Consumption Rate (OCR = pmole O_2_ min^−1^) and ExtraCellular Acidification Rate (ECAR = mpH min^−1^) using a XF‐96 Extracellular Flux Analyzer (Agilent).

### Confocal Microscopy

For frozen sections, DiO‐labeled onion mitochondria were administered to ALI mice via gavage. After 2 h, the mice were euthanized, and the lung tissues were collected. The lung tissues were fixed in periodate‐lysine‐paraformaldehyde (PLP) and dehydrated with 30% sucrose in PBS at 4 °C overnight. The fixed lung tissues were sectioned into 8‐µm thick slices using a Leica cryostat and mounted on slides. Primary antibody anti‐F4/80 antibody (Santa Cruz, sc‐25830) or anti‐TOM‐20 antibody (Proteintech, 11802‐1‐AP) was applied to the lung tissue sections on the slides and incubated at room temperature for 2 h. The sections were then washed three times with PBS, followed by the application of a rabbit anti‐488 secondary antibody. After incubation at room temperature for another 2 h, the sections were washed three times with PBS, and nuclei were stained with 4`,6‐Diamidiono‐2‐phenylindole dihydrochloride (DAPI). The slides were scanned using an Aperio Scan Scope or visualized using a confocal laser scanning microscope.

For in vitro macrophage experiments, sterile coverslips were placed in a 6‐well plate, and macrophages IC‐21 were seeded and incubated overnight in a cell culture incubator. DiO‐labeled onion mitochondria were then added to the 6‐well plate and incubated for an additional 6 h. The coverslips were removed and washed three times with PBS, then placed in a 1% PBST solution and incubated at room temperature for 10 min. TOM‐20 antibody was applied to the coverslips and incubated at room temperature for 2 h. After washing three times with PBS, a 488‐labeled secondary antibody was applied, followed by incubation at room temperature for another 2 h. The coverslips were then washed three times with PBS, mounted on slides, and observed using a laser confocal microscope.

### Flow Cytometry Analysis

Macrophage IC‐21 cells or leukocytes isolated from mouse lungs were incubated with blocking buffer (2% BSA) and subsequently stained for 1 h or overnight at 4 °C with the appropriate fluorochrome‐conjugated antibodies in PBS with 2% FBS. The antibodies against anti‐TOM‐20, anti‐F4/80, anti‐IFN‐γ, and anti‐TNF‐α were purchased from eBioscience and used at a 1:400 dilution. Data were acquired on a BD FACS CantoTM (BD Biosciences, San Jose, CA) and were analyzed using FlowJo software (v10.0, Tree Star Inc., Ashland, OR). Numbers above bracketed lines or boxes in FACS figures indicated percent of positive‐stained cells, and the results of cells stained with an isotype‐matched control antibody were shown in gray color.

### Quantitative Real‐Time PCR for Gene Expression

The quantity of mRNAs was determined with quantitative reverse transcription PCR (q‐PCR). Total RNA (1 mg) was reverse transcribed using SuperScript III reverse transcriptase (Invitrogen), and quantification was performed using SsoAdvanced Universal SYBR Green Supermix (BioRad) with the listed primers (Table S2, Supporting Information). q‐PCR was conducted on a BioRad CFX96 qPCR system, with each reaction performed in triplicate. Data analysis and fold‐change calculations were performed using the comparative threshold cycle (Ct) method. Changes in mRNA expression levels were expressed as fold‐change.

### Western Blotting

Cells and mice were treated as indicated in individual figure legends, and whole cell extracts were prepared in the radioimmunoprecipitation assay (RIPA) buffer (Sigma) with the addition of protease and phosphatase inhibitors (Roche). Proteins were separated by 10% SDS‐PAGE and transferred to polyvinylidene difluoride (PVDF) membranes (Bio‐Rad Laboratories, Inc., Hercules, CA). Dual color precision protein MW markers (BioRad) were separated in parallel. Antibodies were purchased as follows: anti‐CR1L antibody (Cat# BS‐14070R), anti‐phosphor‐DRP1 (Ca# 3455), anti‐DRP1 antibody (Cat# 8570), and anti‐MT‐ND1 antibody (Cat# MP50754‐1), and anti‐GAPDH antibody (Cat# ab9657). The secondary antibodies conjugated to Alex‐594 (A11012) were purchased from Invitrogen (Eugene, OR). The bands were visualized on the Odyssey Imager (LiCor Inc, Lincoln, NE), and band density was analyzed by ImageJ.

### Integrity and Stability of Onion Mitochondria

Mitochondria were isolated from onion and gut epithelial cells and then incubated with gastric fluid or gut fluid at 37 °C for 1 h. After incubation, the mixed solution was centrifuged at 15 000 × g for 15 min at 4 °C, and the pellets were collected. According to the instructions, 200 µL of JC‐1 staining solution was added to the pellets, incubated at 37 °C for 30 min, centrifuged at 15 000 × g for 15 minutes at 4 °C, and washed with PBS three times. The pellets were resuspended in 200‐µL PBS, and fluorescence intensity was measured using a BioTek microplate reader. The red/green fluorescence ratio was calculated to evaluate mitochondrial membrane potential.

### Histological Analysis

For hematoxylin and eosin (H&E) staining, tissues were fixed with buffered 10% formalin solution (SF93–20; Fisher Scientific, Fair Lawn, NJ) overnight at 4 °C. Dehydration was achieved by immersion in a graded ethanol series of 70%, 80%, 95%, and 100% ethanol for 40 min each. Tissues were embedded in paraffin and subsequently cut into ultra‐thin slices (5 µm) using a microtome. Tissues were deparaffinized using xylene (Fisher) and rehydrated by decreasing concentrations of the above ethanol. Tissue sections were stained with H&E, and slides were scanned with an Aperio ScanScope. For frozen sections, tissues were fixed with periodate‐lysine‐paraformaldehyde (PLP) and dehydrated with 30% sucrose in PBS at 4 °C overnight. The sections were incubated overnight at 4 °C with F4/80 (Cat# A18637) and anti‐Tom20 (Cat# 11802‐1‐AP) diluted 1:100. The signal was visualized with the secondary antibodies conjugated to Alexa Fluor‐488 or Alexa Fluor‐594 (Invitrogen), and nuclei were stained with 4′,6‐diamidino‐ 2‐phenylindole dihydrochloride (DAPI). The slides were scanned using an Aperio ScanScope or visualized using confocal laser scanning microscopy (Nikon, Melville, NY).

### Surface Plasmon Resonance (SPR) Analysis

To identify the binding activity of the CR1L and O‐Mit as a lipid manner, SPR experiments were conducted on an OpenSPR (Nicoya, Lifesciences, CA). Experiments were performed on a hydrophobic sensor (Nicoya, Lifesciences). First, the hydrophobic sensor chip was cleaned with 40 mm Octyl β‐*D*‐glucopyranoside (OGP) with a flow rate of 150 µL min^−1,^ and 200 µL of lipid solution in the run buffer (100 µg mL^−1^) was injected on the hydrophobic sensor chip for 10 min until a stable resonance was obtained. After a stable signal was obtained, the CR1L protein (10 nm) was run over the immobilized sensor chip until a stable resonance was obtained.

To identify the binding activity of the ND1 and MDHB, SPR experiments were conducted on an OpenSPR (Nicoya, Lifesciences, CA). Experiments were performed on an amice sensor (Nicoya, Lifesciences). According to the instructions for the amine chip, MDHB was diluted in immobilization buffer to a concentration of 30 µg mL^−1^. A 100 µL EDC solution (0.1 m) and a 100 µL NHS solution (0.1 m) were mixed at a 1:1 ratio, followed by the addition of 200 µL of MDHB solution, which was then injected onto the amine chip. After incubating at room temperature for 20 min, running buffer was injected, and the signal was observed. Once the signal stabilized, ND1 solutions of different fragments were injected onto the immobilized chip, and the signal changes were recorded by OpenSPR.

A negative control test was also performed by injecting onto a blank sensor chip to check for non‐specific binding. The sensograms were analyzed using TraceDrawer kinetic analysis software.

### Preparation of Macrophage IC‐21 Membrane Proteins

The cell plasma membrane proteins were isolated using sucrose gradient ultracentrifugation.^[^ ^45^ ^]^ Macrophage IC‐21 cells (1 × 10^7^) were washed with HES buffer (20 mm HEPES, 1 mm EDTA, 250 mm sucrose, pH = 7.4) three times and then resuspended in 1 mL HES buffer containing protease inhibitors. After disruption using a Dounce homogenizer, the lysates were centrifuged at 196 000 × g for 1 h. The pellets were lysed using 0.5 mL of MBS (25 mm MES, 150 mm NaCl, pH = 6.5) with 0.5% Triton X‐100 and further homogenized with the Dounce homogenizer. The lysates were mixed with an equal volume of 80% w/v sucrose in MBS, and the mixture was carefully dispensed under 2.2 mL of 30% sucrose and 1.4 mL of 5% sucrose solution. Samples were centrifugated at 240 000 × g for 18 h, and the rotor was allowed to stop without braking. The top fraction containing highly enriched membrane protein was collected from the gradients.

### Analysis of Liquid Chromatography–Tandem Mass Spectrometry

The total macrophage IC‐21 membrane proteins (100 µg) isolated with Mem‐PER Plus Membrane Protein Extraction Kit (ThermoFisher) were incubated with O‐Mit (10 mg) for 6 h at 37 °C The O‐Mit‐interacted complex was pull‐down by centrifugation at 12 000 × g for 30 min at 4 °C centrifugation. After washing with PBS, the O‐Mit protein interacting on the macrophage IC‐21 membrane was identified using liquid chromatography–tandem mass spectrometry (LC‐MS/MS) and a previously described method.^[^ ^114^ ^]^ Proteome Discoverer v1.4.1.114 (Thermo) was used to analyze the data collected by MS using the database in Mascot v2.5.1 and SequestHT. Scaffold was used to calculate the false discovery rate using the Peptide and Protein Prophet algorithms.

### Enzyme‐Linked Immunosorbent Assay (ELISA)

Euthanized ALI mice (*n* = 5) were subjected to tracheal exposure via a sterile 22G catheter that was carefully inserted into the trachea. A total of 1 mL of sterile PBS was instilled into the lungs via the trachea, followed by three gentle lavages. The BALF was gently aspirated and collected into sterile centrifuge tubes. The samples were centrifuged at 300 × g for 10 min at 4 °C, and the supernatant was used for cytokine detection by ELISA.

ALI mice (*n* = 5) were euthanized, and blood was collected. After allowing the blood to clot at room temperature for 30 min, the samples were centrifuged at 1000 × g for 10 min at 4 °C. The serum was collected and used for cytokine detection by ELISA.

Cytokines were quantified using ELISA kits (eBioscience) according to the manufacturer's instructions. Briefly, a microtiter plate was coated with anti‐mouse interferon gamma (IFN‐γ), interleukin (IL)‐10, interleukin (IL)‐6, and interleukin (IL)‐1β capture antibodies (eBioscience) at 1:200 at 4 °C overnight. Excess binding sites were blocked with 200 µL of 1× ELISA/ELISPoT Diluent (eBioscience) for 1 h at 22 °C. After washing three times with PBS containing 0.05% Tween 20, the plate was incubated with secondary antibody in blocking buffer for 1 h at 22 °C. After washing three times, avidin conjugated with horseradish peroxidase and substrate were each added sequentially for 1 h and 30 min at 22 °C, respectively. Absorbance at 405 nm was recorded using a microplate reader (BioTek Synergy HT).

### Single‐Strand Gel Shift Assay

To identify the metabolite binding site of ND1, a series of oligonucleotides (oligos) (Eurofins Genomics LLC, KY) specific to the response putative MDHB binding site for the mitochondrial DNA is synthesized.^[^ ^42^ ^]^ The oligos were incubated with 6 nm of MDHB at 37 °C for 30 min following electrophoresis on a 15% native PAGE in 1× TAE buffer. The oligos were stained with ethidium bromide (0.5 mg mL^−1^) and visualized under UV light.

### Statistical Analysis

Unless otherwise indicated, all statistical analyses in this study were performed using SPSS 16.0 software. The data were presented as values with standard deviation (as the mean ± SD). The significance of differences in mean values between two groups was analyzed using the Student's *t*‐test. Differences between individual groups were analyzed via one‐ or two‐way ANOVA. Differences between percentages of bacterial composition were analyzed with a chi‐square test. Differences were considered significant when the *P*‐value was less than 0.05 or 0.01. A *P*‐value greater than 0.05 was considered non‐significant (NS). Animals were randomly assigned to a control group and different experimental condition groups matched for age and sex using simple randomization. Double‐blind studies were used for animal and human subject studies. Unless otherwise indicated, the mice used in the in vivo study were male C57BL/6 mice. Using one‐way ANOVA comparing up to four groups, for a power of 0.7, a large effect size (0.75) and a significance level of 0.05, the minimum sample size needed in each group was 4.992 (rounded = 5).^[^ ^115^ ^]^ The reported “‘n”’ in animal and human studies represented the number of animals and human subjects. Data were representative of at least three independent experiments.

## Conflict of Interest

The authors declare no conflict of interest.

## Author Contributions

Q.X. and Y.T. contributed equally to this work. Q.X., Y.T., and HG.Z. designed the experiments, supervised this study, analyzed, interpreted the data, and prepared the manuscript. Y.H., J.M., L.T., Q.H., H.Q., M.L., Y.Z., L.Z., and X.Z. performed the experiments. Y.J. and M.M. discussed the findings.

## Supporting information



Supporting Information

## Data Availability

The data that support the findings of this study are available from the corresponding author upon reasonable request.
