# Supplementary material for: Onion‐Mitochondria Inhibit Lipopolysaccharide‐Induced Acute Lung Injury by Shaping Lung Macrophage Mitochondrial Function
Source: Adv Sci (Weinh). 2025 Oct 6;12(48):e06107. doi: 10.1002/advs.202506107 (PMC12752597; doi:10.1002/advs.202506107)
Supplement: Supplementary file 1 — Supporting Information [file ADVS-12-e06107-s001.docx]

**Supplementary Figures**

**Fig****ure S1: Characterization of plant-derived mitochondria** (P-Mit). (A) Sucrose-banded mitochondria from onion (O-Mit), garlic (G-Mit) and soybean (S-Mit). (B) Yield of O-Mit, G-Mit and S-Mit. (C) TEM image of O-Mit, G-Mit and S-Mit after embedding and negative staining. Bar scale 600 nm. (D) The lengths of the purified O-Mit, S-Mit, and G-Mit measured using TEM. (E) Zeta potential of O-Mit, G-Mit and S-Mit. (F) The refractive index of O-Mit, G-Mit and S-Mit at different concentrations. (G) Lipids were extracted from purified O-Mit, G-Mit and S-Mit, and mitochondrial lipid composition was analyzed by liquid chromatography–mass spectrometry (LC-MS). (H) The activity of complex 1 from O-Mit, S-Mit, and G-Mit under 37°C and 100°C for 5 min assessed using Complex I Enzyme Activity Microplate Assay Kit (Abcam). Data are representative of three independent experiments (error bars indicate SD). **p < 0.01 (two-tailed t test).

**
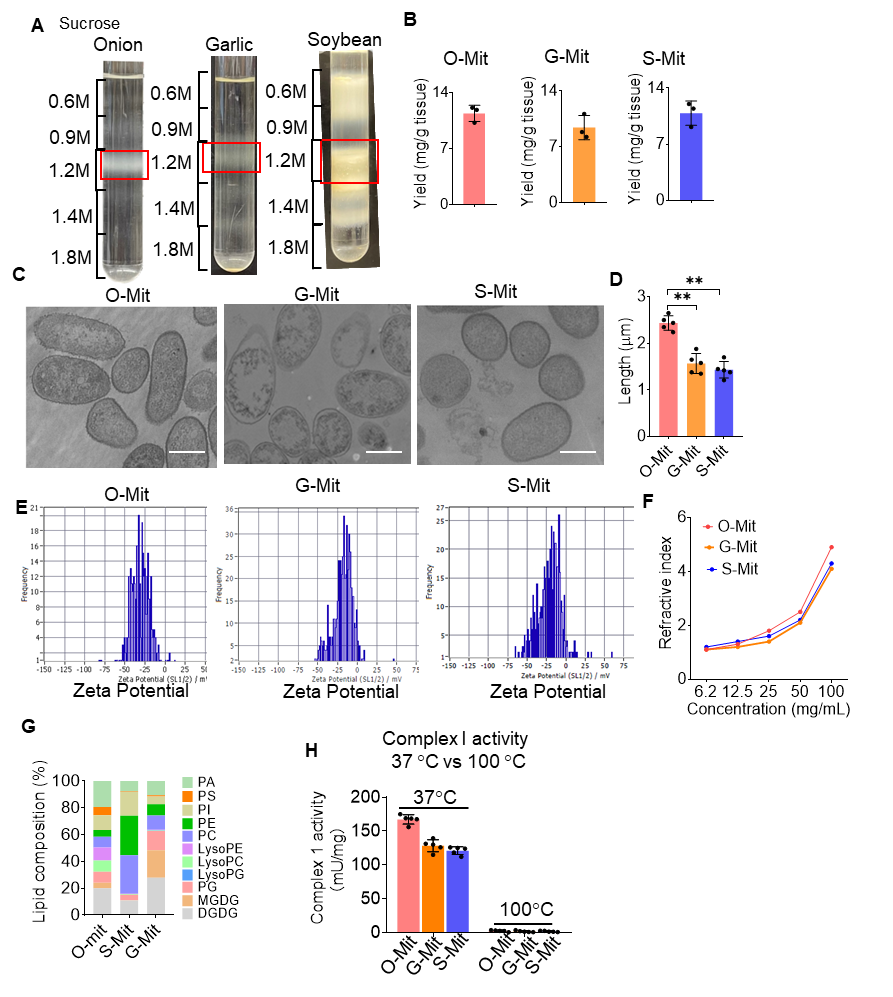
**

**Figure S2 Effect of P-Mit in LPS-induced inflammation in vitro in different doses.** (A-F) After induction with LPS, macrophage IC-21 cells were treated with various concentrations of O-Mit, G-Mit, and S-Mit for 6 hours. The cell medium was then collected and the levels of the cytokines IFN-γ and IL-1β were measured by ELISA. Data are representative of three independent experiments (error bars indicate SD). vs PBS, *p < 0.05, **p < 0.01, ***p < 0.001 (two-tailed t test).


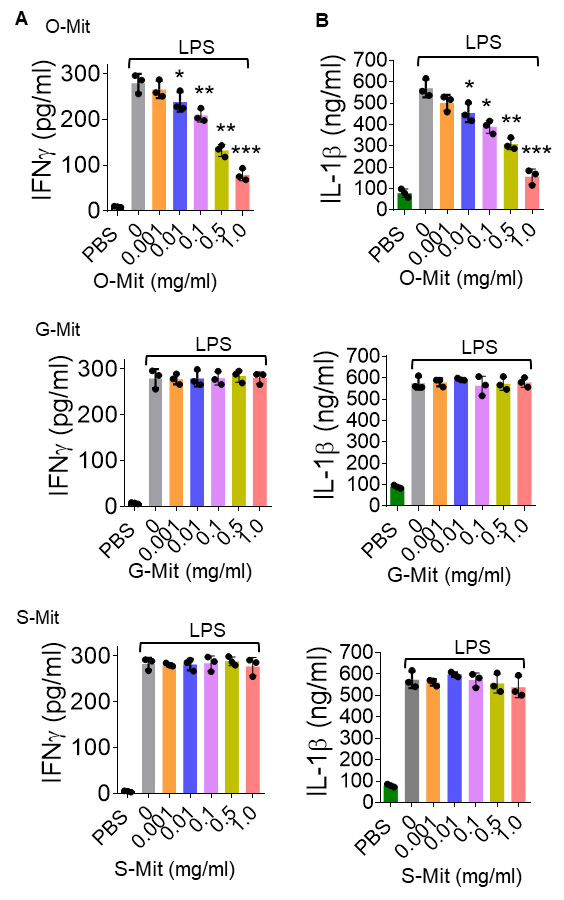


**Figure S3 Integrity and stability of onion-derived mitochondria (O-Mit) in gastric fluid and gut fluid.** (A, B) Isolated mitochondria from onion (O-Mit) and gut epithelial cells (E-Mit) were incubated with PBS, gastric fluid, or gut fluid at 37°C for 1 hour. Mitochondrial potential was assessed by measuring the red/green fluorescence ratio of JC-1 (A) and mitochondrial oxygen consumption rate (OCR) (B). The results showed that in O-Mit there was no change in the JC-1 red (590 nm)/green (529 nm) fluorescence ratio or the OCR in gastric and gut fluids compared to PBS. In contrast, mitochondria isolated from gut epithelial cells (E-Mit) showed a significant decrease in both the JC-1 red/green fluorescence ratio and the OCR in gastric and gut fluids compared to PBS. (C) Mitochondria isolated and purified from onion or gut epithelial cells were incubated with gastric and gut fluid at 37°C for 1 h followed by analysis of mitochondrial gene nad1 expression using PCR. The results showed that mitochondrial gene nad1 in O-Mit remained stable in both gastric and gut fluids, whereas gene nad1 in E-Mit was significantly reduced. (D) O-Mit and O-Mit derived lipids were incubated with gut and gastric fluid at 37°C for 1 hour. Lipids were then extracted and separated using thin-layer chromatography (TLC). The TLC results showed that O-Mit was stable in both gut and gastric fluids, whereas no detectable lipids were found after incubation of O-Mit-derived lipids with gut and gastric fluid. Data are representative of three independent experiments (error bars indicate SD). *p < 0.05, **p < 0.01 (two-tailed t test).

**
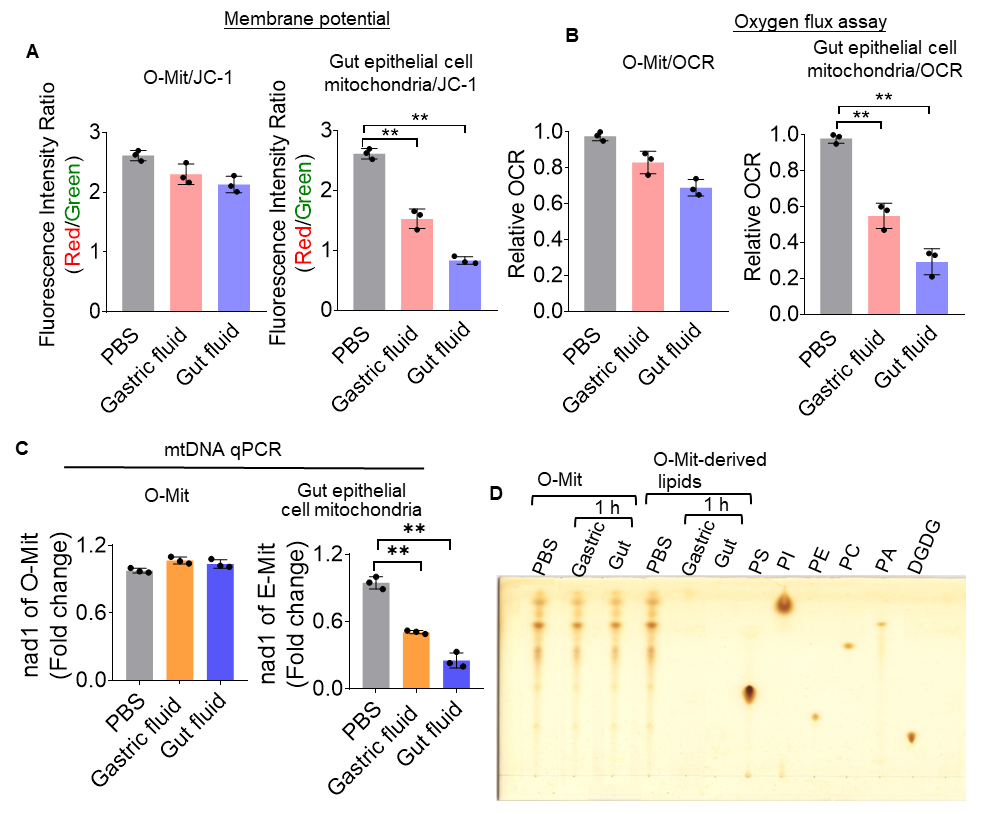
**

**Figure S4. Impact of P-Mit on the Induction of Intestinal Regulatory T Cells (Tregs).** (A, B) ALI mice were orally gavaged with PBS, O-Mit, G-Mit, or S-Mit. Six hours post-gavage, the small intestine was harvested for leukocyte isolation. Regulatory T cells (Tregs) were identified by flow cytometry using anti-CD4 and anti-FoxP3 antibodies. The expression of IL-10 and TGF-β within FoxP3⁺CD4⁺ T cells was analyzed (left panel), and quantified (right panel). (C) ALI mice were orally administered DiO-labeled O-Mit. After 2 hours, colocalization of O-Mit/DiO with F4/80⁺ macrophages or CD4⁺ T cells in the small intestine was assessed by confocal microscopy. Scale bar: 20 μm. (D) Flow cytometric analysis of TGF-β and reactive oxygen species (ROS) in F4/80⁺ macrophages isolated from the small intestine. (E) F4/80⁺ macrophages and FoxP3⁺CD4⁺ Tregs were sorted from ALI mice by FACS. Macrophages were treated with O-Mit for 6 hours, and the conditioned medium was collected and used to incubate Tregs in the presence or absence of a TGF-β neutralizing antibody (1 μg/mL) for 6 hours. IL-10 and TGF-β levels in Treg cell lysates (10 μg protein) were measured by ELISA. Data are representative of three independent experiments. Error bars indicate standard deviation (SD). Statistical analysis was performed using Chi-square and t-tests: *p < 0.05, **p < 0.01, ***p < 0.001.

**
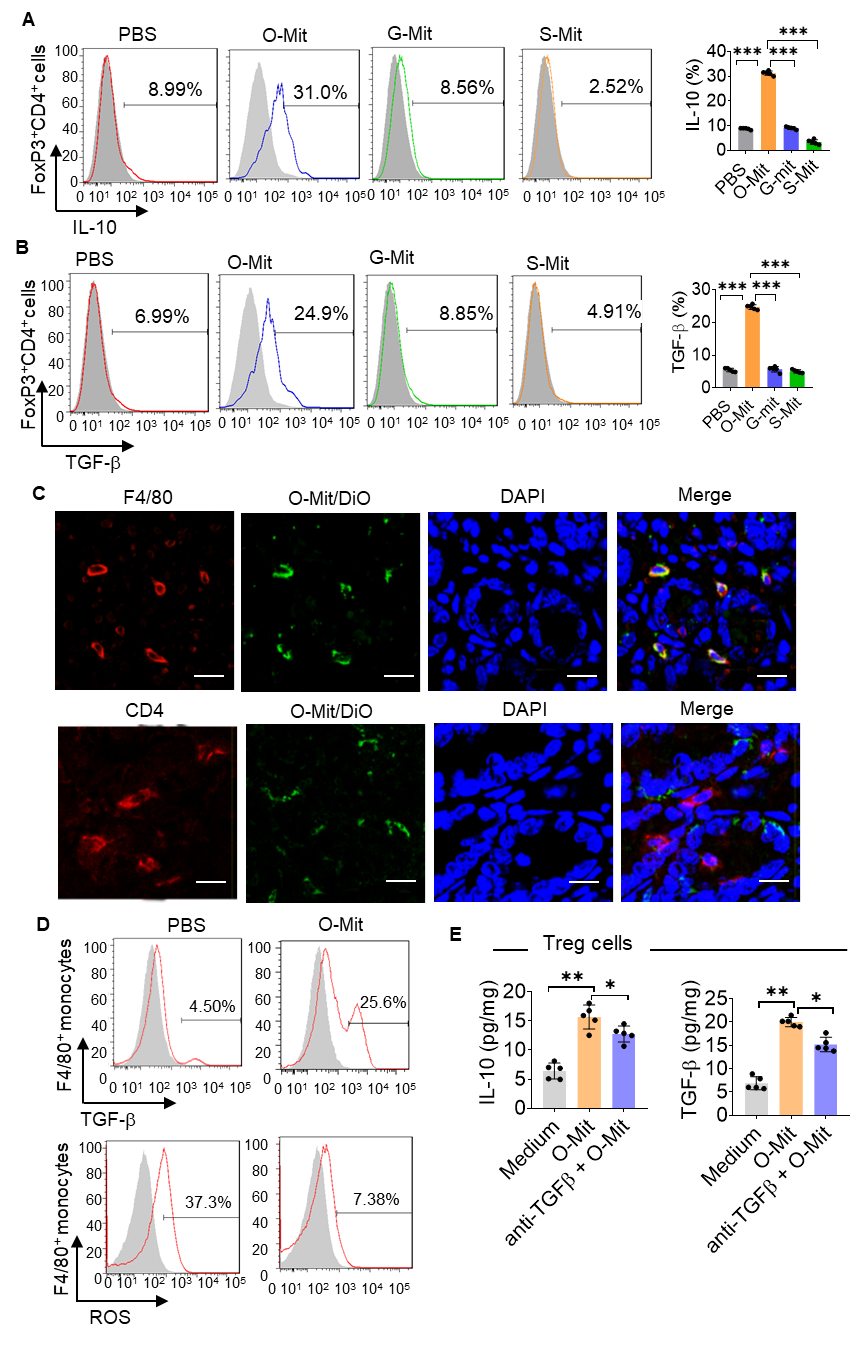
**

**Figure S5: Distribution of gavageI -given onion-mitochondria (O-Mit) in B6 mice.** (A) ALI B6 mice were gavaged with P-Mit/DiR (O-Mit/DiR, S-Mit/DiR, or G-Mit/DiR), and organs were collected 6 hours later. The distribution of P-Mit/DiR in organs were evaluated using a fluorescence imaging system. (B) Fluorescence intensity analysis further confirmed that the lungs showed the highest fluorescence signal among all groups, with statistically significant differences. (C) ALI mice were gavaged with O-Mit/DiR and serum samples were collected at various time points to measure fluorescence intensity. The fluorescence signal in the serum peaked at 2 h post-gavage and then gradually declined. (D) Purified O-Mit was further separated into mitochondria of different sizes using a sucrose density gradient. The isolated mitochondria were observed by TEM, and the mitochrondrial lengths were analyzed. The results demonstrated that mitochondria of different sizes were successfully separated from purified O-Mit, with statistically significant differences. Bar scale: 600 nm. (E) ALI mice were gavaged with large-sized and small-sized O-Mit/DiR. Six hours later the mice were euthanized and fluorescence intensity in lung and liver tissues was detected using a fluorescence imaging system. (F) Naïve B6 mice were gavaged with free DiR dye and P-Mit/DiR. Six hours later the mice were euthanized and lung and liver tissues were scanned using a fluorescence imaging system. Data are representative of three independent experiments (error bars indicate SD). *p < 0.05, **p < 0.01, ***p < 0.001 (two-tailed t test).

**
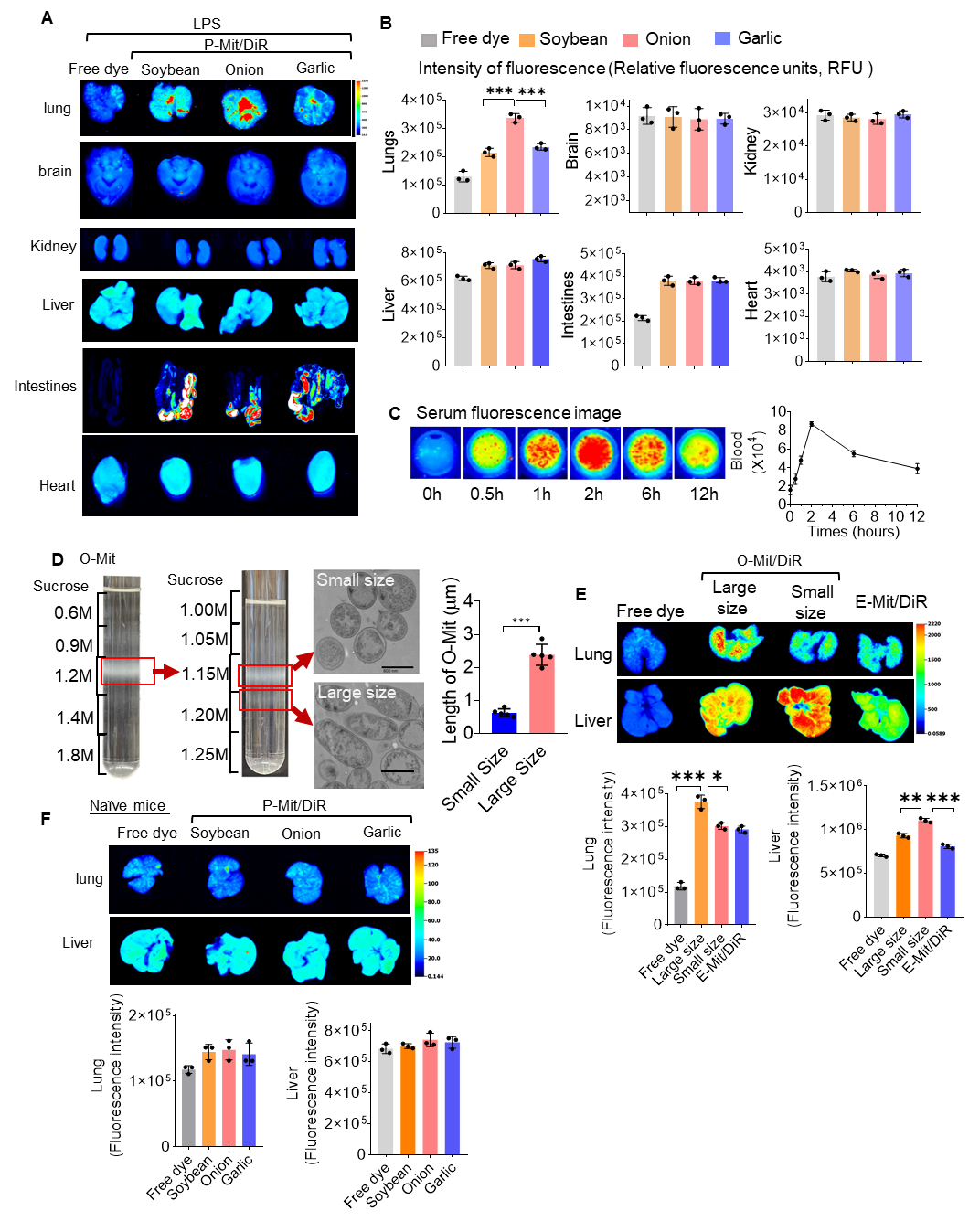
**

**Figure S6: Integrity and stability of onion-derived mitochondria (O-Mit) in lungs.** (A) ALI B6 mice oral gavage with O-Mit. Lung bronchoalveolar lavage fluid (BALF) collected at 1 and 2 hours after gavage followed by mitochondria isolation. Western blot was performed to analyze the level of the plant mitochondrial protein IDH3. Input represents the mount of O-Mit gavage. (B) PCR was used to assess the level of O-Mit mtDNA gene nad1. (C) ALI mice were orally gavaged with O-Mit/DiO, and lung BALF was collected at 1 and 2 hours after gavage followed by mitochondria isolation. Flow cytometry (FACS) was used to analyze the fluorescence intensity of O-Mit/DiO. (D) ALI mice were gavaged with O-Mit labeled with mitochondrial membrane potential dye JC-1 and lung BALF was collected followed by mitochondria isolation. mitochondrial membrane potential analysis using O-Mit/JC-1 fluorescence indicator at 590 nm (red, polarized) and 529 nm (green, depolarized) (left panel). The ratio of JC-1 red/green represents the activity of O-Mit (right panel). Data are representative of three independent experiments (error bars indicate SD). *p < 0.05, **p < 0.01, ***p < 0.001, ns; not significant (two-tailed t test).

**
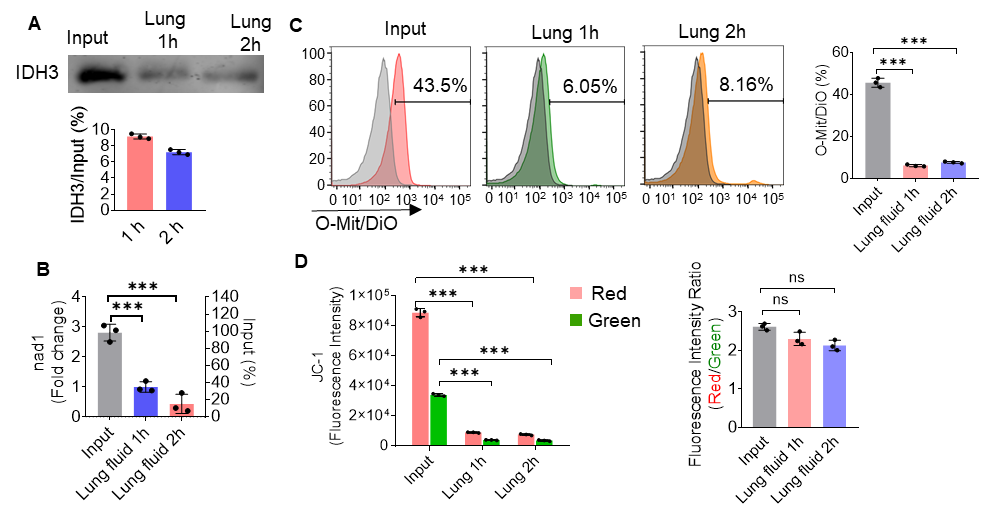
**

**Figure S7. The influence of O-Mit on macrophage phagocytosis.** (A) Macrophages were incubated with LPS (10 μg/ml) and the phagocytic activity was assessed using the Vybrant Phagocytosis Assay Kit. The results showed that LPS enhanced the phagocytic activity of the macrophages. (B) Six hours after ALI mice were orally gavaged with O-Mit/DiI, the lungs were collected, embedded, and sectioned. Epithelial cells were labeled with anti-EpCAM and nuclei were stained with DAPI. Confocal microscopy revealed no colocalization of O-Mit/Dil with epithelial cells (400X magnification, scale bars, 200 μm). (C) ALI mice were orally gavaged with E-Mit/DiO and after 6 hours, the mice were euthanized, and lung tissues were collected and processed into monocytes. Macrophages were labeled with anti-F4/80 and flow cytometry (FACS) analysis showed that macrophages had taken up E-Mit/DiO. (D) LPS was incubated with macrophages and E-Mit was added to the culture. The supernatant was collected, and the levels of IL-6 and IFN-γ in the supernatant were measured by ELISA. The results indicated that E-Mit did not inhibit the secretion of cytokines IL-6 and IFN-γ. Statistical analysis was performed using the Chi-square test: *p < 0.05, **p < 0.01, ***p < 0.001. Data are representative of three independent experiments (error bars indicate SD). *p < 0.05, **p < 0.01, ns; not significant (two-tailed t test).


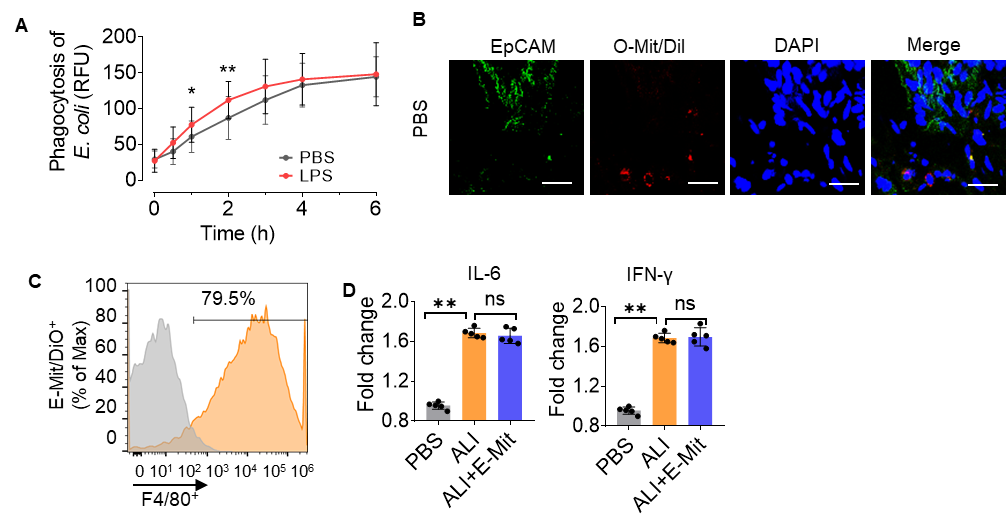


**Figure S8. O-Mit Protein and Nucleic Acid Are Not Required for O-Mit–Mediated Inhibition of LPS-Induced Acute Lung Injury (ALI)**. (A) O-Mit was incubated with protease or RNase at 37°C for 30 minutes. Protein degradation was assessed by 15% SDS-PAGE followed by Coomassie Brilliant Blue staining, and nucleic acid digestion was evaluated by 2% agarose gel electrophoresis. (B) Membrane potential of T-Mit treated with protease (in regular maintenance buffer [MB] or modified MB) or RNase was analyzed using the JC-1 assay. Statistical comparison versus untreated O-Mit: p < 0.01, ns = not significant (Chi-square test). (C) ALI mice were orally gavaged with O-Mit pre-treated with protease or RNase. Lung tissue sections were stained with hematoxylin and eosin (H&E). Original magnification: ×20; scale bar: 100 μm. Arrows indicate inflammatory cell infiltration in the alveolar cavity and interstitial space, as well as thickening of the alveolar wall. (D–E) Levels of cytokines IFN-γ, IL-1β, IL-6, and IL-10 in bronchoalveolar lavage fluid (BALF) (D) and serum (E) were measured by ELISA in ALI mice from different treatment groups. Data are representative of three independent experiments. Error bars indicate standard deviation (SD). Statistical analysis was performed using two-tailed t-tests: p < 0.01, ***p < 0.001


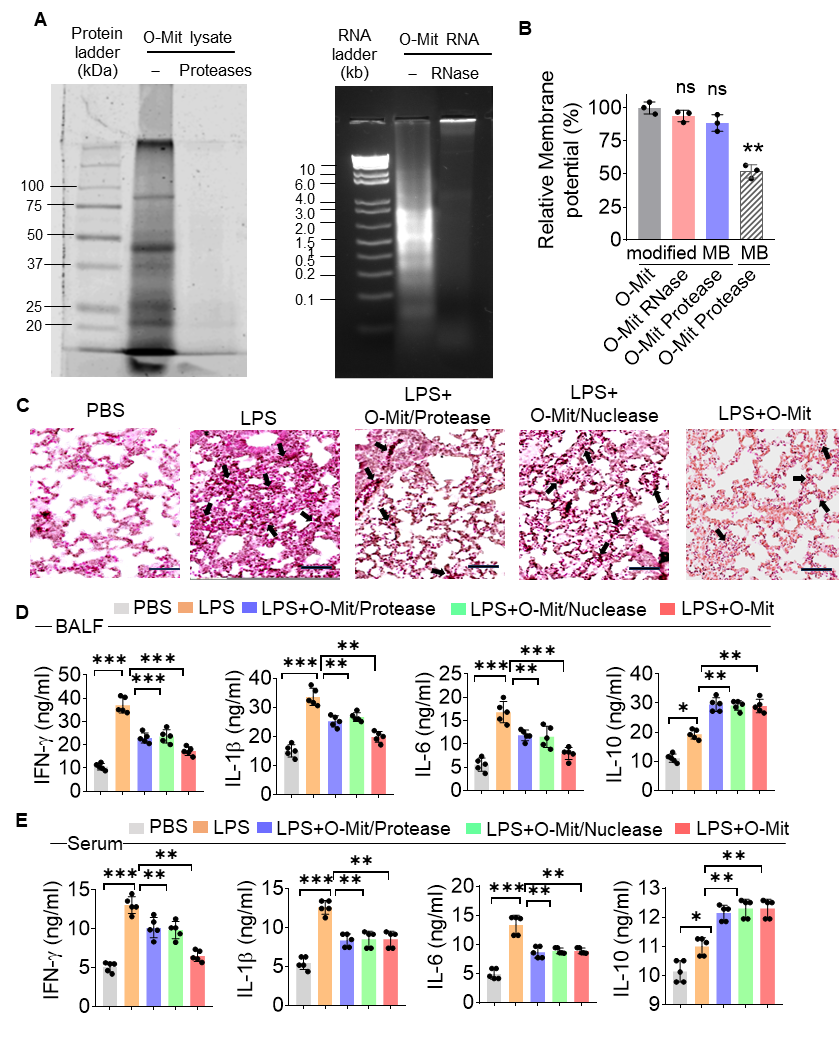


**Figure S9: Analysis of O-Mit toxicity.** (A) Different concentrations of O-Mit were added to macrophages and cell viability was measured at various time points. The results indicated that O-Mit had no effect on the growth of macrophages. (B) Mice were orally gavaged with different concentrations of O-Mit and blood samples were collected to assess ALT (left) and AST (right) levels. The results showed that liver function was not affected by O-Mit administration. Data are representative of three independent experiments (error bars indicate SD, two-tailed t test).


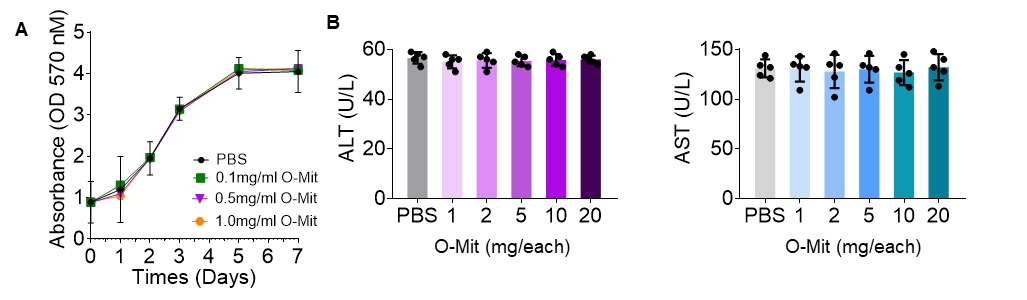


**Supplementary Tables**

**Table S1 List of O-Mit small molecule metabolites identified by LC-MS**

| **Name** | **Reference(m/z)** | |
| --- | --- | --- |
| (19R,25S)-22,25,26-Trihydroxy-22-oxido-16-oxo-17,21,23-trioxa-22lambda~5~-phosphahexacosan-19-yl (9Z)-9-hexadecenoate | | 497144.64 |
| (2E)-decenoic acid | | 145790.19 |
| (2E,4E)-N-Isobutyl-2,4-octadecadienamide | | 115097.84 |
| (3aR,4R,5R,6aS)-5-Hydroxy-4-[(1E,3S)-3-hydroxy-1-octen-1-yl] hexahydro-2H-cyclopenta[b]furan-2-one | | 78402.67 |
| (3beta,4beta,5alpha,6alpha,15alpha,22E,24R,25R)-Ergost-22-ene-3,4,5,6,8,14,15,25,26-nonol | | 31661.84 |
| 1-(4-Aminobutyl) urea | | 228512.51 |
| 1-linoleoyl-sn-glycero-3-phosphoethanolamine | | 360129.07 |
| 2-[3-carboxylato-3-(methylammonio)propyl]-L-histidine dizwitterion | | 148191.47 |
| methyl 3,4-dihydroxybenzoate (MDHB) | | 73584.25 |
| 3-O-beta-D-galactosyl-sn-glycerol | | 270934.59 |
| 6-Acetamido-2-oxohexanoic acid | | 64617.92 |
| Aceglutamide | | 13629.94 |
| Acetylcadaverine | | 182816.79 |
| ALA-PRO | | 131622.07 |
| Cormethasone acetate | | 49013.39 |
| D,L-alpha-Aminoisobutyric acid | | 1269374.41 |
| Diethylpyrocarbonate | | 250521.21 |
| DL-p-Hydroxyphenyllactic acid | | 23855.766 |
| his-gln | | 51155.39 |
| histidinol | | 124382.15 |
| H-Met(O)-OH | | 10654.11 |
| Inosine | | 1091807.69 |
| Leucylasparagine | | 176145.09 |
| Leucyl-leucyl-norleucine | | 11911.06 |
| Leu-Gly-Gly | | 708377.68 |

**Table S2 List of primers used in q-PCR**

| **Name** | **Forward** | **Reverse** |
| --- | --- | --- |
| mIL-6 | GAGAGGAGACTTCACAGAGGATAC | GTACTCCAGAAGACCAGAGG |
| mIL-1α | ATCAGTACCTCACGGCTGCT | TGGGTATCTCAGGCATCTCC |
| mIL-10 | TTTGAATTCCCTGGGTGAGAA | GGAGAAATCGATGACAGCGC |
| mIFN-γ | CTTTGCAGCTCTTCCTCATGGCTGTTTCTG | TGACGCTTATGTTGTTGCTGATGGCCTG |
| mND1 | CCTAACACTCCTCGTCCCCATTCT | GGGTCAGGCTGGCAGAAGTAATCAT |
| mNAD1 | TGCCATCTACCTTCTTCAACCTCACC | TTAGCATTGTAGTAGGTTGAGATTTTGGACG |
| GAPDH | GGTCGGTGTGAACGGATTTG | GGAGTCATACTGGAACATGTAG |
| O-Mit  NAD1 | TGGGTGTACTGGACCTCTAAT | AAGGAGGGAGTTTGCTTTACC |
